# Study of Iron Piperazine-Based Chelators as Potential Siderophore Mimetics

**DOI:** 10.3390/ph12040160

**Published:** 2019-10-23

**Authors:** Pauline Loupias, Isabelle Dechamps-Olivier, Laurent Dupont, Pierre Vanlemmens, Catherine Mullié, Nicolas Taudon, Anne Bouchut, Alexandra Dassonville-Klimpt, Pascal Sonnet

**Affiliations:** 1 AGIR, EA 4294, UFR de Pharmacie, Université de Picardie Jules Verne, 1 rue des Louvels, 80037 Amiens, France; pauline.loupias@etud.u-picardie.fr (P.L.); pierre.vanlemmens@u-picardie.fr (P.V.); catherine.mullie@u-picardie.fr (C.M.); anne.bouchut@sattnord.fr (A.B.); alexandra.dassonville@u-picardie.fr (A.D.-K.); 2 ICMR, UMR 7312, 3 Avenue du Maréchal Juin, 51100 Reims, France; isabelle.dechamps@univ-reims.fr (I.D.-O.); laurent.dupont@univ-reims.fr (L.D.); 3 Unité de Toxicologie Analytique, Institut de Recherche Biomédicales des Armées, 91223 Brétigny-sur-Orge, France; nicolas.taudon@gmail.com

**Keywords:** *Pseudomonas* group, siderophore mimetics, chelators, iron, piperazine

## Abstract

Gram-negative bacteria’s resistance such as *Pseudomonas aeruginosa* and the *Burkholderia* group to conventional antibiotics leads to therapeutic failure. Use of siderophores as Trojan horses to internalize antibacterial agents or toxic metals within bacteria is a promising strategy to overcome resistance phenomenon. To combat the *Pseudomonas* sp, we have synthesized and studied two piperazine-based siderophore mimetics carrying either catecholate moieties (**1**) or hydroxypyridinone groups (**2**) as iron chelators. These siderophore-like molecules were prepared in no more than four steps with good global yields. The physicochemical study has highlighted a strong iron affinity since their pFe values were higher than 20. **1** possesses even a pFe value superior than those of pyoverdine, the *P. aeruginosa* endogenous siderophore, suggesting its potential ability to compete with it. At physiological pH, **1** forms mainly a 2:3 complex with iron, whereas two species are observed for **2**. Unfortunately, the corresponding Ga(III)-**1** and **2** complexes showed no antibacterial activity against *P. aeruginosa* DSM 1117 strain. The evaluation of their siderophore-like activity showed that **1** and **2** could be internalized by the bacteria.

## 1. Introduction

Among Gram-negative bacteria, *Pseudomonas aeruginosa* and *Burkholderia pseudomallei* are particularly worrying. *P. aeruginosa* is often involved in nosocomial infections (6.2% of all hospital-acquired infections) [1,2]. *B. pseudomallei*, formerly classified as *P. pseudomallei*, causes melioidosis (Whitmore disease) which prevails in tropical climates. Cases were also reported in the U.S. [3,4]. The most common form of this disease, pulmonary melioidosis, resembles to tuberculosis and is particularly recalcitrant to therapy with a high relapse rate [5]. The research team, AGIR, is implicated in the development of new ways to combat Gram-negative bacteria. In particular, the *Pseudomonas* group is targeted due to its resistance to antibiotics via a lack of membrane permeability or efflux, leading to therapeutic failure and requires new antibiotic therapies.

Using iron transport systems is a promising strategy to overcome the lack of membrane permeability by restoring the activity of conventional antibiotics [6,7]. Iron is an essential metal for, playing key structural and chemical roles in protein cofactors (heme and Fe-S clusters) [8]. The insolubility of the ferric ion under biological conditions (10^−18^ M at neutral pH) makes it a vital nutriment in cellular homeostasis. During the infectioN′s establishment, the situation is exacerbated because serum proteins and the liver sequestrate the free Fe(III) [9]. In an effort to scavenge iron from both natural environmental and infection settings, bacteria have developed pathways to synthesize, secrete, and retrieve small molecule chelators called siderophores [10]. These chelators display a very high affinity for ferric iron which can be measured by the pFe value under physiological conditions. The pFe is the negative logarithm of the free ferric iron in solution at pH 7.4 for defined concentration of ligand (10^−5^ M) and iron (10^−6^ M) [11,12]. This very high affinity for ferric irons enables siderophores to drive the dissolution of insoluble salts in the environment or to strip off ferric iron from host sequestering proteins. Depending on their chemical functional groups, siderophores can be divided into three main groups: (i) catecholates or phenolates as enterobactin, (ii) hydroxamates as deferoxamine (DFO), and (iii) α-hydroxycarboxylates as staphyloferrin A. Most often, the same oxygen-donor group is represented two or three-fold into linear or cyclic platforms to form tetradentate (3:2/siderophore-Fe(III) complexes) or hexadentate ligands (1:1/siderophore-Fe(III) complexes). They can also be mixed in pyoverdine (Pvd) or pyochelin (Pch), the two main siderophores of *Pseudomonas* [13]. Each bacterium possesses its own outer membrane receptor (OMR), specific to each siderophore. This way, they can uptake siderophores produced by other species. *P. aeruginosa* secures its iron acquisition by internalization of Pvd and Pch, recognized respectively by the FpvA (or FpvB) and FptA [14]. This strain is also able to use exogenous iron chelators through the expression of different OMRs such as FecA (for ferric citrate), PfeA and PirA (for enterobactin), FoxA and FiuA (for DFO and ferrichrome), FemA (for mycobactin) and ChtA (for rhizobactin and aerobactin) [6].

**Figure 1:**
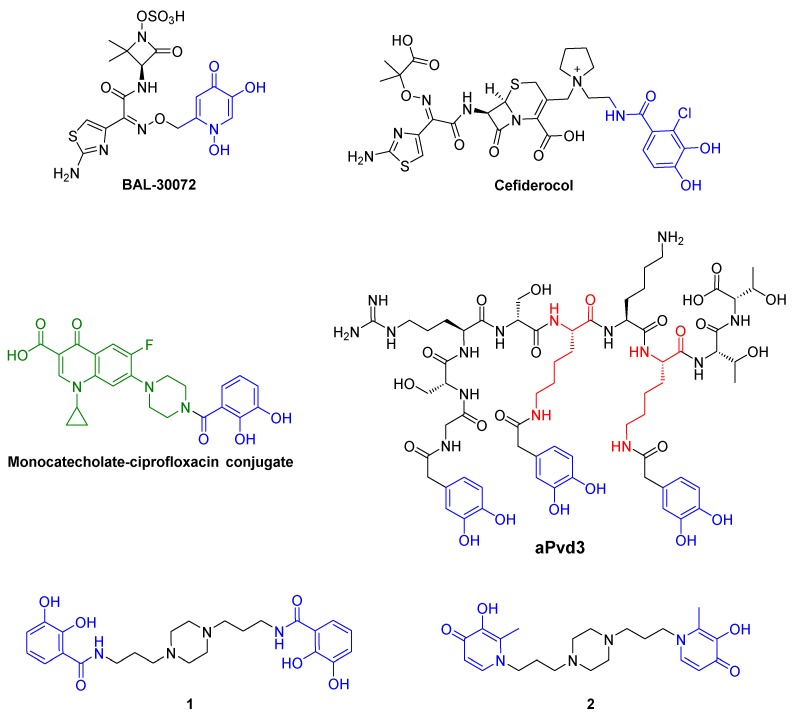
BAL-30072, cefiderocol, monocatecholate–ciprofloxacin conjugate, **aPvd3** and synthesized siderophore mimetics **1** and **2**.

These OMRs are different entry routes for toxic complexes and antibiotics using the “Trojan Horse Strategy”. These two concepts were reported in the antipseudomonal strategy. In 2008, DFO-Ga(III) complex was described as a potential therapeutic agent against *P. aeruginosa* [15]. More recently, the great activities of two siderophore-antibiotic conjugates, BAL 30,072 [16,17,18,19] and cefiderocol [20,21], against resistant strains of *P. aeruginosa* were described (Figure 1). They possess either a 1,3-dihydroxypyridin-4-one group, a catechol bioisostere, or a catechol group, as siderophore moiety that are recognized by the OMR of this strain [17,19].

We have already reported the synthesis and the antipseudomonal activity of a series of catechol-ciprofloxacin conjugates with the monocatecholate–ciprofloxacin conjugate as the lead compound (Figure 1) [22]. Later, we have highlighted the Pvd analog aPvd3 (pFe = 21.4) carrying three catechol groups as an alternative to Pvd (pFe = 27) for iron uptake by *P. aeruginosa* [23]. As piperazine possesses good pharmacokinetic properties, piperazine derivatives are often used for therapeutic purposes [24,25,26]. Especially in the antimicrobial area, this heterocycle nucleus is found in a lot of antibacterial quinolone medicine such as ciprofloxacin or antifungal drugs like posaconazole [27]. However, many recent studies reported the antimicrobial potential of *N*-substituted piperazines [26]. Additionally, particular compounds such as 1-(1-naphtylmethyl)-piperazine are known to act as efflux pump inhibitors helping to restore antibiotic activity on resistant strains [28]. Moreover, this piperazine scaffold is easily functionalizable and allows broad access to derivatives of biological interest. Thus, we decided to use the 1,4-bis(3-aminopropyl)piperazine to graft two identical iron ligands, catechol or 3-hydroxypyridin-4-one [29], to obtain a rapid access to the siderophore mimetics, **1** and **2** respectively.

Herein, we report the synthesis, the physicochemical properties and the *P. aeruginosa* siderophore-like activity of the piperazine-based chelators **1** and **2**.

## 2. Results and Discussion

### 2.1. Siderophore Mimetics **1** and **2** Synthesis

**Figure 2:**
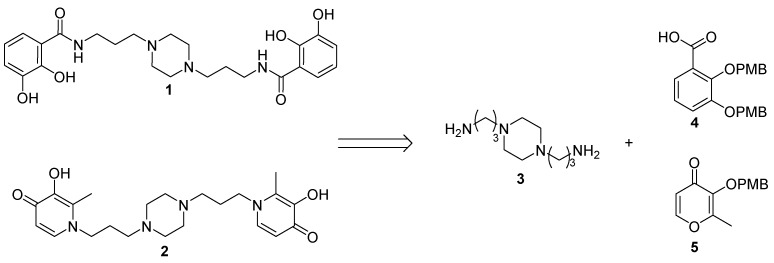
Retrosynthesis of the iron chelators **1** and **2**.

The synthesis of two iron chelators **1** and **2** relies on a coupling reaction between, respectively, the bidentate ligand **4** or **5** precursors and the 1,4-bis(3-aminopropyl)piperazine **3** (Figure 2).

#### 2.1.1. Synthesis of the Bidentate Ligand Precursors **4** and **5**

**Figure 3:**
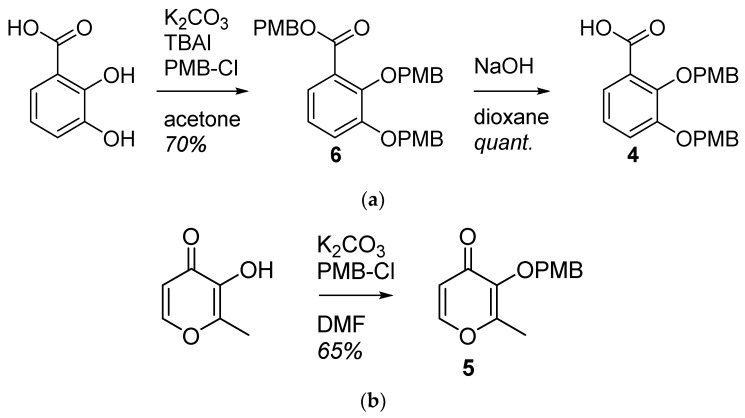
Synthesis of the bidentate ligands precursors (**a**) **4** is obtained from the 2,3-dihydroxybenzoic acid; (**b**) **5** is obtained from the 3-hydroxy-2-methyl-4*H*-pyran-4-one (maltol).

The 2,3-dihydroxybenzoic acid was protected in the presence of potassium carbonate, tetrabutylammonium iodide and *p*-methoxybenzylchloride. **6** was saponified with sodium hydroxide to afford the bidentate ligand **4** in two steps with a 70% yield. The maltol was protected using potassium carbonate and *p*-methoxybenzylchloride with a 65% yield (Figure 3).

#### 2.1.2. Synthesis of the Iron Chelators **1** and **2**

**Figure 4:**
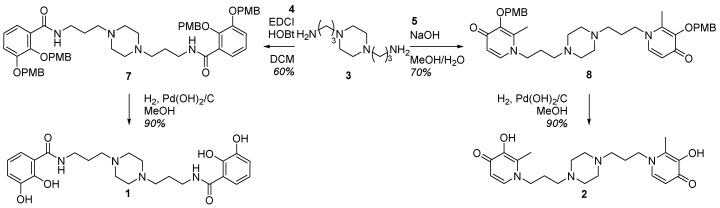
Synthesis of the iron chelators bearing catecholate (**1**) or hydroxypyridinone (**2**) moieties.

The bidentate ligands precursors **4** and **5** were synthesized in order to be coupled with the 1,4-bis(3-aminopropyl)piperazine **3**. A peptide coupling has been carried out between the precursor **4** and **3**, giving **7** with a 60% yield. A condensation of the protected maltol **5** and the piperazine **3** gave **8**, with a 70% yield. The common hydrogenation step was performed using an H-cube system generating hydrogen by electrolysis of water. **1** and **2** were, respectively, obtained in a 4 and 3-step synthesis with a 38% and 41% global yield (Figure 4).

### 2.2. Physicochemical Studies

After synthesis, physiochemical studies have been performed on both siderophore mimetics **1** and **2**.

The complexing ability towards Fe(III) of siderophores is evaluated from the pFe determination, which represents the fraction of uncomplexed Fe(III) at physiological pH (pH = 7.4), using standardized conditions ([ligand] = 10^−5^ M, [Fe] = 10^−6^ M, T = 25 °C, I = 0.1 M). The expressed pFe (pFe = −log[Fe]), represents the overall chelating ligand ability when taking into account all the complex species present in the medium and the ligand affinity. The higher the value of this parameter, the higher the chelating ability of the ligand is. The ligands we developed are often difficult to solubilize in pure water, thus restricting the study of their equilibriums with Fe(III) in solution. The simplest alternative then is to use mixed solvents. Thereby, complexations of Fe(III) cations with siderophore mimetics were studied in water/DMSO mixtures by potentiometry and UV-visible spectrophotometry. More specifically, the stoichiometry and the overall stability constants of all soluble species were determined. The potentiometry is based on the comparison of the neutralization curves of the ligand alone and in the presence of the metal with fluctuating ratios R = [L]/[M], where [L] and [M] represent the analytical concentrations of the ligand and the metal introduced in solution, respectively. This method is particularly suitable to study ligands which have weak acid-base properties. Base moieties usually constitute complexation sites for the metal ions. The complexation reactions result in the release of additional protons comparatively to that of the neutralization of the ligand alone. The solution has a much more acidic character when the complexes are stable. Most often, the analysis of the titration curves allows to determine the stoichiometry of the predominant complexes. The solutions are also analyzed by mass spectrometry with electrospray ionization to facilitate and confirm the qualitative analysis of titration curves. UV-visible spectra of ligand solutions in the presence of metal are recorded, and the spectra refinement allows the determination of the formation constants of the complexes and the specific electronic spectrum of the absorbing species. Information about the geometry and the coordination modes of the metal cations can be deduced from these spectra. The agreement between the values of the formation constants determined by potentiometry, and those determined by UV-visible spectrophotometry, constitutes a validation of the proposed chemical model for each studied system. After study of the species distribution diagrams, the biologically active forms of the complexes at physiological pH can be identified, and the pFe value determined.

#### 2.2.1. System H+/1(2)

**Figure 5:**
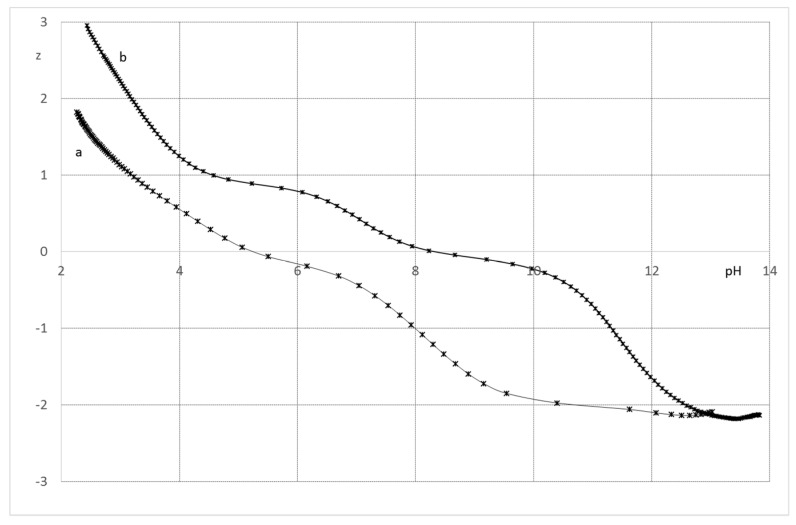
Average charge of the ligands as a function of pH; KCl 0.1 mol.L^−1^; T = 298 K. (**a**) **1**; (**b**) **2**.

The ligand **1** in its fully protonated state, may release potentially six protons, two from the piperazine function and four from the two catechols. The neutralization curve was converted as a z versus pH curve taking into account that the neutral form of the ligand is LH_4_. The curve, performed in the absence of metal, shows an initial decrease of z, followed by a plateau between pH 5 and 7 for a z value of −0.2 (Figure 5). This is ascribed to the neutralization of the protons bound to piperazine. The negative value of z at the plateau indicates that the neutralization of the first hydroxyl of the catechol functions is effective in this pH range. The value of z, higher than −2 for a pH greater than 10, indicates that the second hydroxyl of the catechol functions remain protonated throughout the titration.

The ligand **2** has six protonable groups: two pyridinium, two piperazines and two hydroxyls. The neutral form is LH_2_ with the two hydroxyl groups remaining protonated. The z versus pH curve of the ligand shows three deprotonation steps. The neutralization curve of the ligand shows that at the beginning of the titration nearly 50% of the pyridine groups are protonated. The plateau between pH 4 and 6 at a z value of 1, corresponds to the neutralization of the pyridinium ions and one of the protons bound to the piperazine cycle. The second step of deprotonation, corresponding to a plateau between pH 7.5 and 9 with a z value of 0, corresponds to the loss of the second protons bound to the piperazine cycle. The decrease of z value above pH 8, leading to a negative value of z, corresponds to the neutralization of the two hydroxyl groups of the ligand (Figure 5).

Acidity constants of the **1** and **2** ligands were estimated on the basis of the global protonation constants. The protonation constant corresponds to the β_01h_ and is determined by refinements of neutralization curves of the ligands in the absence of metal (Table 1). The low values of the estimated standard deviation σ_mlh_ of the β_mlh_ constants, and of the global standard deviation σ_mlh_ of the β_mlh_ constants (<0.95), are indicative of the validity of the models for the fitting procedure of **1** and **2** titrations.

For **1**, the pKa_1_ and pKa_2_ values match well with those for the deprotonations of the nitrogen atoms of the piperazine cycle, whereas pKa_3_ and pKa_4_ correspond to the deprotonation to the first hydroxyl of the catechol function. This allocation is corroborated by the analysis of the spectrophotometric titration. Indeed, the spectra of **1** remain insensitive to pH variations within the 2–4.5 pH range and show only one characteristic π-π* transition centered at 305 nm. This transition is due to the absorbance of the fully protonated catechol function. Above pH 4.5, the increase in pH causes a gradual shift of this transition to higher wavelengths, and the appearance of new transitions at 330 nm, whose intensity increases with pH. This transition is ascribed to the deprotonation of the hydroxyl groups of the catechol [30].

For **2**, the pKa_1_ and pKa_2_ values match well with the value found for the deprotonations of the nitrogen atoms of the pyridinic groups, while pKa_3_ and pKa_4_ are ascribed to the deprotonation of the nitrogen atoms of the piperazine cycle [31]. The higher value of pKa_4_ compared to pKa_3_ is indicative of strong electrostatic interactions between the two nitrogen atoms of piperazine moieties. On the basis of study made on deferiprone, we can confidently attribute pKa_5_ and pKa_6_ to the neutralization of hydroxyl groups bound to the aromatic cycle [32].

#### 2.2.2. System Fe(III)/1(2)

Extensive research on siderophores and iron chelators allows the pH-based monitoring of the evolution of catechol-iron complexes by UV-vis spectrophotometry. The analysis is on the location of the ligand-to-metal charge transfer band (LMCTs) [30,33,34]. The 1:1 (Fe(III)(cat)); 1:2 (Fe(III)(cat)_2_) and 1:3 (Fe(III)(cat)_3_) species are produced at low (below pH 4.5), intermediate (pH 7.5) and alkaline pH (above pH 9.5), respectively, and show intense specific LMCT transitions in the visible region. For Fe(III)(cat) complexes, LMCT bands are centered around 720 nm, while Fe(III)(cat)_2_ and Fe(III)(cat)_3_ transitions are located around 570 and 470 nm, respectively.

**Figure 6:**
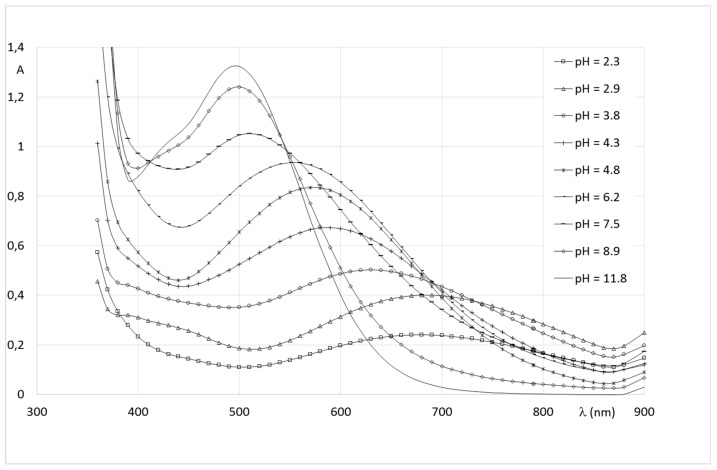
Spectrophotometric titration of Fe(III)-**1** solutions.

The spectral characteristic of Fe(III)-**1** complexes are similar to those of iron-catechol ones and indicate a progressive involvement of the catechol groups in the coordination of the metal, with the increase of pH (Figure 6). Indeed, beyond pH 4, and the LMCT for the Fe(III)-**1** solution is close to 700 nm, and is easily related to the formation of a complex with one catechol group bound to the metal. Mass spectrum of Fe(III)-**1** indicate the formation of a 1:1 complex detected in positive mode at *m*/*z* = 526.15 corresponding to [Fe + L − 2H]^+^. The isosbestic point between pH 5 and 8 corresponds to an equilibrium between the 1:1 and 2:2 complexes. The 2:2 complex shows a transition centered at 580 nm, corresponding to the coordination of two catechols from distinct ligands, on two Fe(III) centers. The transitions shift between pH 7.5 and 9 towards higher energy is ascribed to the formation of the 2:3 species by addition of a third ligand on the metal centers. This complex shows a transition at 500 nm, characteristic of a Fe(cat)_3_ chromophore [30,33,34]. The 2:3 species was detected at high pH by mass spectrometry in negative mode as a discharged species at *m*/*z* = 882 [2Fe + 3L + 4Na + 2DMSO − 6H].

**Figure 7:**
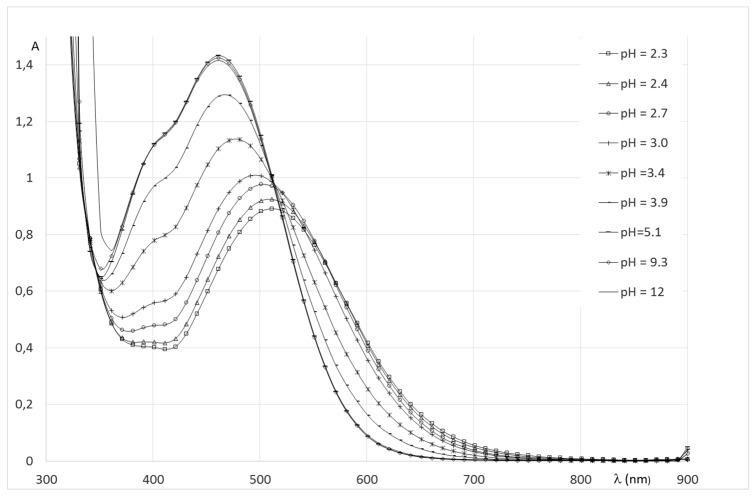
Spectrophotometric titration of Fe(III)-**2** solutions.

The spectral evolution of Fe(III)/**2** solutions with pH, (Figure 7) present similar features to those as reported by Nurchi et al. on the system Fe(III)-deferiprone [32]. The absorbance properties of solutions show that the coordination of oxygen atoms of the aromatic groups is effective from the beginning of titration. From pH 2 to 2.7, the spectra show an intense transition at 510 nm, growing with pH, corresponding to a molar absorbance greater than 3000 mol^−1^.dm^+3^.cm^−1^. On the basis of the results, this transition ascribed to an LMCT transition, generated by the coordination of two ligands, to form a chromophore Fe(O_ar_)_4_(O_w_)_2_ around the metal center. Mass spectra recorded in the pH range indicate that it corresponds to a complex of stoichiometry 2:2. Indeed, in this pH range, only one complex species is detected in positive mode at *m*/*z* = 470.1. The simulation of the isotopic cluster shows the unique formation of a 2/2 discharged complex in this pH range corresponding to Fe_2_L_2_H_−4_ [2Fe + 2L − 4H^+^]^2+^. The spectrophotometric titration shows an isosbestic point between pH 2.7 and 4.2 that reflects equilibria between two chromophores. However, the mass spectra of solutions in this pH range do not show additional species, which seems to indicate that the isosbestic points correspond to an equilibrium between two deprotonated forms of a 2:2 species, unlike the results of Nurchi et al. which propose the coordination of a third molecule of deferiprone around the metal center. The mass spectrum recorded at pH 9 shows two additional peaks with very low intensity at *m*/*z* = 1355 and 1377, corresponding to an adduct of a 2:3 species [2Fe + 3L − 5H]^+^ and [2Fe + 3L − 6H + Na]^+^ formed to a small extent.

Refinements of potentiometric titrations allow the determination of stability constants (β_mlh_) for the Fe(III)-**1** and Fe(III)-**2** complexes. To this purpose, the features of spectrophotometric titrations, and the results of mass spectrometry were highly considered to define the best chemical model for both systems (Table 2).

**Figure 8:**
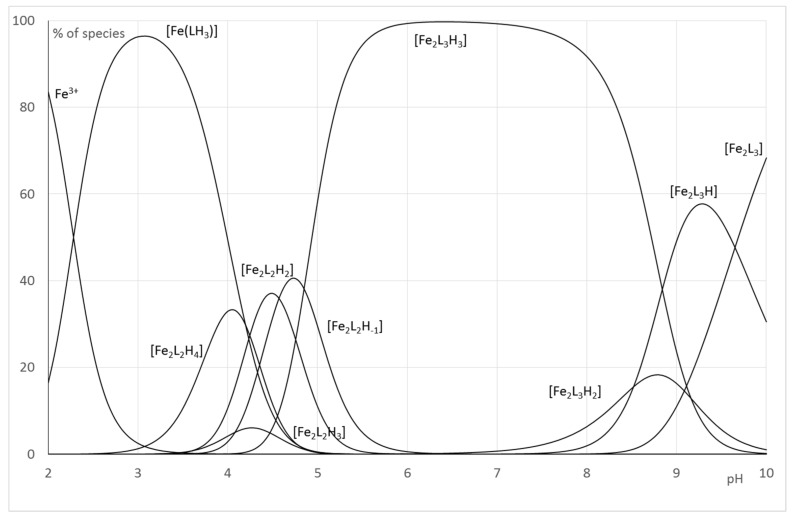
Distribution curves for the Fe-**1** systems (CL = 1.10^−3^ mol.L^−1^; Cm = 2.10^−4^ mol.L^−1^).

In order to make these equilibrium constants more explicit, the distribution curves of the various species were plotted as a function of pH for R = 5 (Figure 8).

For the Fe(III)-**1** system, the speciation curve underlines the formation of complexes above pH 2. The distribution curves agree with the spectrophotometric titration and underline a maximum for the formation of Fe(**1**) complexes at pH 3.2 (corresponding to the maximum of the formation of the species Fe(LH)^+^), and the maximum for the formation of Fe_2_(**1**)_2_ is located at pH 4.6 and corresponds to an equilibria between Fe_2_L_2_H_−2_ and Fe_2_L_2_H_−3_, whereas Fe_2_(**1**)_3_ complexes predominate above pH 5. In all cases, for 1:1 and 2:2 complexes, the successive deprotonation steps correspond to the loss of the protons of the second hydroxyl groups of the catechol function.

**Figure 9:**
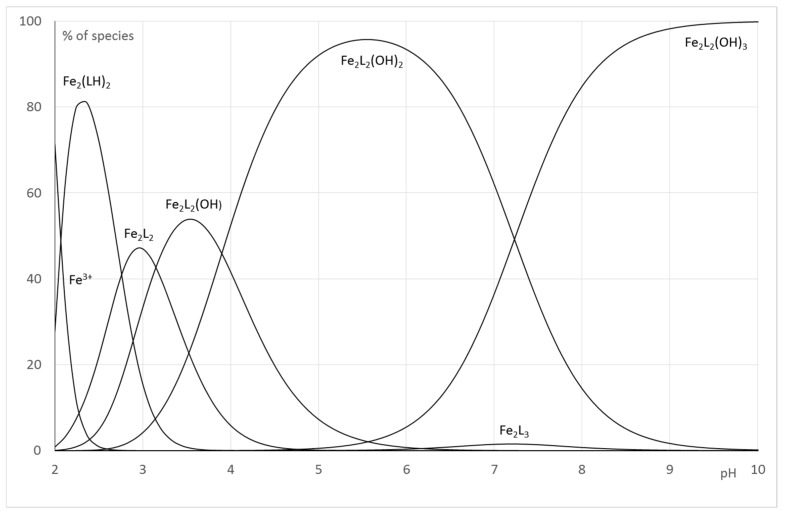
Distribution curves for the Fe-**2** systems (CL = 1.10^−3^ mol.L^−1^; Cm = 2.10^−4^ mol.L^−1^).

For the Fe(III)-**2**, the best refinements, in accordance with the interpretation of spectrophotometric titrations and mass spectra recorded at variable pH, confirm the formation of 2:2 species on the whole pH range. The change of chromophore beyond pH 3 corresponds to the deprotonation to the neutralization of nitrogen atoms of pyridine groups and aromatic hydroxyl groups to form Fe_2_L_2_. Above pH 3, the further deprotonations species do not induce modification of the absorbance properties of Fe(III)-**2** solutions. It is reasonable to consider that it concerns a loss of protons from coordinated water molecules. Consequently, the formation constant β_22-1,_ β_22-2_ and β_22-3_ correspond mainly to stoichiometry Fe_2_L_2_(OH)_n_. The Fe_2_L_3_ which has been detected in small extent by mass spectrometry was included in our refinements (Figure 9).

**Figure 10:**
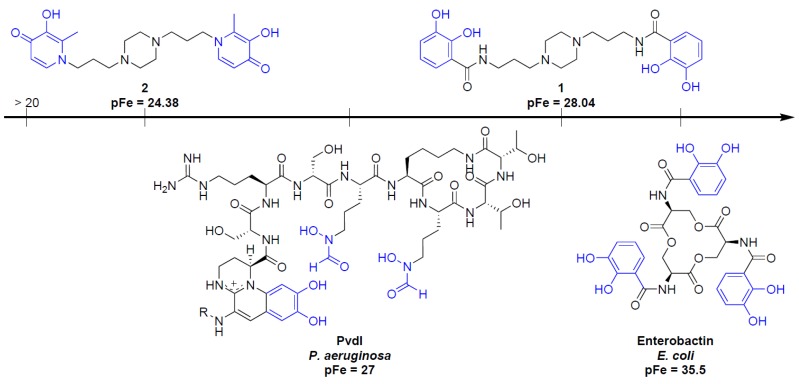
Comparison of pFe values of **1**, **2**, PvdI and enterobactin.

In designing iron chelators for clinical applications, metal selectivity and ligand–metal complex stability are of paramount importance. The most suitable way to compare the Fe(III) chelating ability between ligands **1** and **2**, is to determine pFe. Unlike stability constants, pFe takes into account the effects of ligand basicity, ligand protonation, and metal hydrolysis as well as differences in metal-ligand stoichiometry. The pFe was derived from ligand protonation and Fe-complex formation constants. For clinically relevant conditions, pFe values are typically calculated in pure water at pH 7.45 for total ligand and Fe concentrations equal to 10^−5^ and 10^−6^ mol.L^−1^, respectively. Here, taking into account the extended pH range due to DMSO, the free Fe(III) concentration was calculated at pH 8.44 (see the Experimental Section). The value of pFe obtained for Fe(III)-**2** and Fe(III)-**1** are, respectively, 24.38 and 28.04. Compared to the strongest iron chelator, enterobactin (pFe = 35.5), **1** and **2** possess inferior Fe(III) complexing abilities. In both cases, the values of pFe (>20) meet the criteria as an iron chelation therapy agent. Nonetheless, only **1** shows a pFe superior to Pvd (pFe = 27), the endogenous siderophore of *P. aeruginosa* [35,36,37], as shown in Figure 10.

### 2.3. Antibacterial Activities

The antibacterial activity of the siderophore mimetics **1** and **2** was measured on *P. aeruginosa* DSM 1117 in both cation-supplemented Mueller-Hinton (MH) Broth, as advised by the Clinical and Laboratory Standards Institute (CLSI) [38], and in Succinate Minimum Medium (SMM), a medium virtually deprived of iron. The Minimum Inhibitory Concentration (MIC) was measured with a concentration range of 0.25 to 512 mg·L^−1^ and was determined as the lowest concentration at which wells remain clear. Ciprofloxacin was used as the control and displayed MICs values of 0.5 mg·L^−1^. No intrinsic antibacterial activity was observed for **1** and **2** since the MICs measured are greater than 512 mg·L^−1^ for both. The cytotoxicity was also evaluated on the Hep-G2 cell line, a human liver cancer cell line. **1** and **2** did not show toxicity at concentrations greater than 100 mmol·L^−1^ (Table 3).

### 2.4. Siderophore-Like Activities

**Figure 11:**
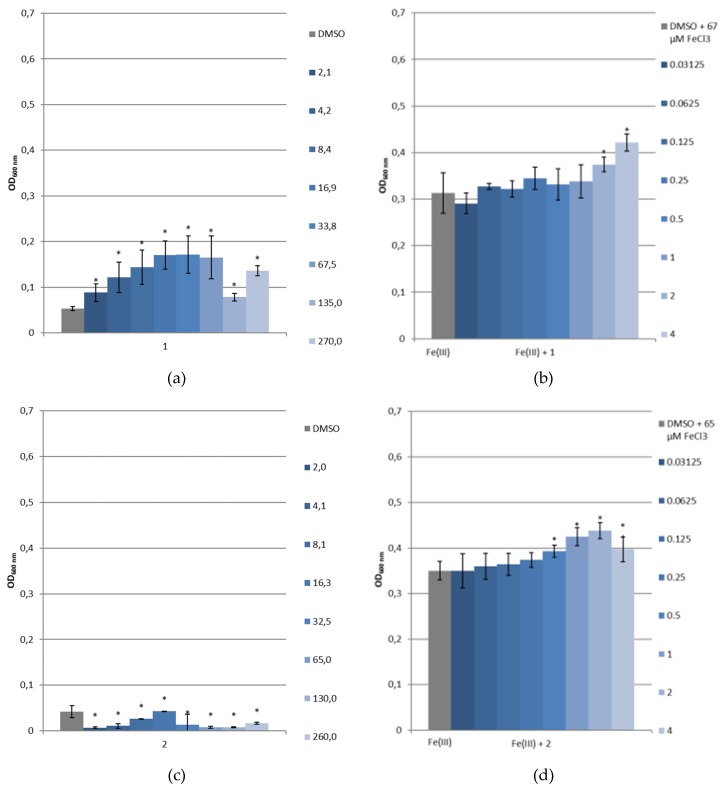
Growth of *P. aeruginosa* PAD07 in SMM (**a**) without ferric iron and with **1**, (**b**) with ferric iron and with **1**, (**c**) without ferric iron and with **2** or (**d**) with ferric iron and with **2**. Values given in the caption at the right of the histograms indicate for (**a**,**c**) the [**L**] concentration in μM and for (**b**,**d**) the [**L**]/[Fe(III)] molecular ratios. *: *p* < 0.05 (vs. control, Mann–Whitney test).

**Figure 12:**
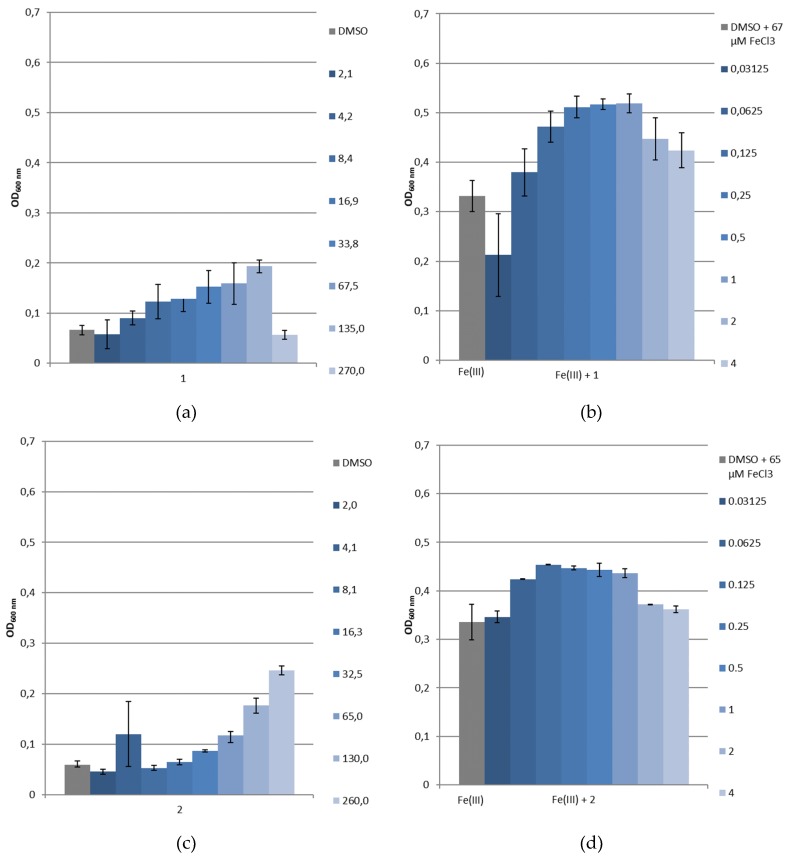
Growth of *P. aeruginosa* PAO1 in SMM (**a**) without ferric iron and with **1**, (**b**) with ferric iron and with **1**, (**c**) without ferric iron and with **2** or (**d**) with ferric iron and with **2**. Values given in the caption at the right of the histograms indicate for (**a**,**c**) the [**L**] concentration in μM and for (**b**,**d**) the [**L**]/[Fe(III)] molecular ratios.

A first screening of the siderophore mimetics has been carried out using growth experiments with *P. aeruginosa* PAO1 as a reference strain, (Deutsche Sammlung für Mikroorganismen, Braunschweig, Germany), and its pyoverdine and pyochelin-double deficient mutant *P. aeruginosa* PAD07 [39]. SMM was used to evaluate the siderophore-like activity of **1** and **2** without and with the addition of a known amount of ferric iron. Controls, without the tested compounds, show the natural bacterial growth under these latter conditions. The microbial development was followed by measuring the optical density at 600 nm (OD_600nm_) [40]. Figure 11a,c and Figure 12a,c represent the *Pseudomonas* straiN′s growth in function of the siderophore mimetics concentration ([**L**]), and Figure 11b,d and Figure 12b,d show their growth in function of the ratio [**L**]/[Fe(III)]. For **1**, as demonstrated by physicochemical studies, the predominant complex at physical pH is Fe_2_L_3_. Thus, we worked assuming that above a 1.5 ratio, all the free Fe(III) was chelated. At pH 7, two species coexist for **2**, Fe_2_L_2_(OH)_2_ and Fe_2_L_2_(OH)_3_, corresponding to a ratio equal to 1.

As shown in Figure 11a, without supplementation of iron, the addition of **1** (2.1–270 μM) only slightly changed the growth of *P. aeruginosa* PAD07. With iron, the growth of this strain with **1** (**1**/Fe(III):0.03125 to 4, 0.32 < OD_600nm_ < 0.42) is superior to the control (67 μM of FeCl_3_, OD_600nm_ = 0.31, Figure 11b). In Figure 11c, the increasing addition of **2** (2.03–260 μM), without supplementation of iron, limits the growth of *P. aeruginosa* PAD07. As observed for **1**, with iron, the addition of **2** (**2**/Fe(III):0.03125 to 4, 0.35 < OD_600nm_ < 0.44) allows a higher growth than the control (65 μM of FeCl_3_, OD_600nm_ = 0.35, Figure 11d). The growth stimulating activity of the two iron chelators are quite comparable. The increasing addition of **1** (2.1–270 μM) or **2** (2.03–260 μM), without supplementation of iron, results in dose-dependent growth of *P. aeruginosa* PAO1 (Figure 12a,c). This bacterial growth could be explained by a Pvd production. Indeed, we have observed a dose dependent yellow–green coloration, characteristic of the uncomplexed Pvd. In iron supplemented SMM, the bacterial growth of *P. aeruginosa* PAO1 is facilitated by the addition of **1** (**1**/Fe(III):0.03125 to 4) or **2** (**2**/Fe(III):0.03125 to 4) (Figure 12b,d). In both cases, the bacterial growth is greater than those of the control (65 or 67 μM of FeCl_3_, OD_600nm_ = 0.33). This growth is slightly higher for **1** (0.38 < OD_600nm_ < 0.52) compared to **2** (0.35 < OD_600nm_ < 0.45). Moreover, the growth of *P. aeruginosa* PAO1 is slightly greater than those of *P. aeruginosa* PAD07 with the addition of **1** (Figure 11b vs. Figure 12b) or **2** (Figure 11d vs. Figure 12d).

These different results suggest that **1** and **2** could be internalized by the bacteria. The difference of bacterial growth for *P. aeruginosa* PAO1 compared to *P. aeruginosa* PAD07, in iron supplemented SMM, with the addition of **1** or **2**, could be due to a Pvd’s additional effect in iron transport. The greater growth effect of **1** compared to **2** could be partly explained in terms of an iron chelator property: the pFe value of **1** (28.04) is close to Pvd’s (27), while the pFe value of **2** (24.38) is lower than those of Pvd’s (27).

According to these data, we used these iron piperazine-based chelators **1** and **2** to internalize, within *P. aeruginosa*, toxic metal such as gallium. Consequently, Ga(III)-**1** and **2** complexes were synthetized (see Supplementary Data), and their antibacterial activities were measured on *P. aeruginosa* DSM 1117 in both MH and SMM, such as previously described in 2.3 (Table 4). Unfortunately, no intrinsic antibacterial activity was observed for Ga(III)-**1** and **2** complexes since the MICs measured are greater than 512 mg.L^−1^ for both. The cytotoxicity was also evaluated on the Hep-G2 cell line, a human liver cancer cell line. Ga(III)-**1** and **2** complexes did not show toxicity at concentrations greater than 100 mmol.L^−1^.

## 3. Materials and Methods

### 3.1. Synthesis

All commercially available products were used without further purification unless otherwise specified. All solvents were dried via literature procedures when reactions required anhydrous conditions, or used without further purification. Flash column chromatography purifications were carried out on silica gel (Kieselgel 60, 40–63 μm, 230–400 mesh ASTM, Merck, Darmstadt, Germany). Analytical TLC were performed on precoated silica gel 60 F254 plates (Merck) and the compounds were visualized under a UV light (254 nm), and with ethanolic phosphomolybdic acid. Hydrogenation was performed using the hydrogen generation system of the H-Cube^®^ Mini (ThalesNano, Budapest, Hungary). Melting points (mp) were determined on a Stuart SMP3 apparatus and reported uncorrected. Infrared spectra were recorded on a Jasco FT/IR-4200 spectrometer system coupled to an ATR module. The wavelengths √ obtained are expressed in cm^−1^. ^1^H and ^13^C NMR spectra were recorded using Bruker 400 MHz spectrometer. Chemical shifts are reported in parts per million (δ, ppm) and the signals are quoted as s (singlet), bs (broad singlet), d (doublet), bd (broad doublet), dd (doublet of doublet), dt (doublet of triplet), t (triplet), bt (broad triplet), q (quartet), bq (broad quartet), and m (multiplet). J values are given in Hertz. Signals assignments were made using HMBC, HSQC, COSY, and NOESY experiments when necessary. LC-HRMS analyses were performed on an ACQUITY UPLC H-Class system (Waters-Micromass, Manchester, UK) coupled with a SYNAPT G2-Si Q-TOF hybrid quadrupole time-of-flight instrument (Waters-Micromass, Manchester, UK), equipped with an electrospray (ESI) ionization source (Z-spray), and an additional sprayer for the reference compound (Lock Spray, Torrance, CA, USA) heated at 50 °C. For liquid chromatography coupled with mass spectrometry (LCMS), UV chromatograms and mass spectra were obtained from a Shimadzu LCMS-2020 system, at 190 nm, and by positive ESI-MS interface (detection mode: scan, interface voltage: tuning file, DL voltage: 100 V, Q-array DC: 40 V, Q-array RF: 40 V). The gradient elution was performed on a Phenomenex Kinetex^®^ HPLC C18 column using an injection volume of 1–2 μL, and a mobile phase composed of water/acetonitrile (solvent A/solvent B) with 0.1% formic acid (98:2 during 2 min, 55:45 during 2 min, and 45:55 during 3 min with a flow of 0.3 mL/min at 40 °C).

*4-methoxybenzyl 2,3-bis((4-methoxybenzyl)oxy)benzoate (**6**)*

To a solution of 1 g (6.48 mmol, 1 equiv.) of 2,3-dihydroxybenzoic acid in 30 mL of acetone was added 2 g (14.5 mmol, 2 equiv.) of potassium carbonate and 1 g (3.24 mmol, 0.5 equiv.) of tetrabutylammonium iodide. The mixture was stirred at 25 °C for 1 h. 3.6 mL (25.92 mmol, 4 equiv.) of 4-methoxybenzylchloride was added and stirred under reflux overnight. The resulting solution was concentrated under reduced pressure. The residue was purified by flash chromatography (Cyclohexane:AcOEt 4:1) to yield a white powder (1.93 g, 70%). mp: 90 °C (*litt*. 108 °C [41]) √_max_: 3066, 3032, 1706, 1610, 1581, 1509, 1241, 1174, 1020 cm^−1^; ^1^H NMR (400 MHz, CDCl_3_): δ 3.79 (s, 3H, OCH_3_), 3.80 (s, 3H, OCH_3_), 3.82 (s, 3H, OCH_3_), 4.95 (s, 2H, CH_2_), 5.04 (s, 2H, CH_2_), 5.25 (s, 2H, CH_2_), 6.77 (d, *J* = 8.6 Hz, 2H, H_ar_), 6.87 (d, *J* = 8.7 Hz, 2H, H_ar_), 6.90 (d, *J* = 8.6 Hz, 2H, H_ar_), 7.05 (t, *J* = 8.0 Hz, 1H, H_ar_), 7.12 (dd, *J* = 1.7, 8.2 Hz, 1H, H_ar_), 7.19 (d, *J* = 8.6 Hz, 2H, H_ar_), 7.31–7.36 (m, 5H, H_ar_) ppm; ^13^C NMR (100 MHz, CDCl_3_): δ 55.4 (OCH_3_), 55.4 (OCH_3_), 55.5 (OCH_3_), 66.8 (OCH_2_), 71.3 (OCH_2_), 75.4 (OCH_2_), 113.7 (C_ar_), 114.1 (C_ar_), 118.3 (C_ar_), 123.0 (C_ar_), 123.9 (C_ar_), 127.1 (C_ar_), 128.3 (C_ar_), 128.8 (C_ar_), 129.5 (C_ar_), 129.8 (C_ar_), 130.3 (C_ar_), 130.4 (C_ar_), 148.5 (C_ar_O), 153.0 (C_ar_O), 159.5 (COCH_3_), 159.7 (COCH_3_), 159.7 (COCH_3_), 166.5 (C = O) ppm; HRMS (ES+): exact mass calculated for C_31_H_30_O_7_Na: 537.1889. Found 537.1895.

*2,3-bis((4-methoxybenzyl)oxy)benzoic acid (**4**)*

To a solution of 500 mg (0.97 mmol, 1 equiv.) of **6** in 12 mL of dioxane was added 4.9 mL (9.7 mmol, 10 equiv.) of sodium hydroxide 2M. The reaction was stirred at 25 °C for 3 days. The mixture was concentrated under reduced pressure. The residue was dissolved in water and an aqueous solution of 1M HCl was added dropwise until pH 2. The precipitate was filtrated and washed with hexane to yield a white powder (1.93 g, quant.). mp: 110 °C (*litt*. 119 °C [41]) √_max_: 3004, 1579, 1512, 1304, 1246, 1173 cm^−1^; ^1^H NMR (400 MHz, DMSO-*d_6_*): δ 3.74 (s, 3H, OCH_3_), 3.77 (s, 3H, OCH_3_), 4.89 (s, 2H, CH_2_), 5.10 (s, 2H, CH_2_), 6.84 (d, *J* = 8.6 Hz, 2H, H_ar_), 6.97 (d, *J* = 8.6 Hz, 2H, H_ar_), 7.12 (t, *J* = 7.8 Hz, 1H, H_ar_), 7.19 (dd, *J* = 1.7, 7.8 Hz, 1H, H_ar_), 7.27 (d, *J* = 8.6 Hz, 2H, H_ar_), 7.33 (dd, *J* = 1.7, 7.8 Hz, 1H, H_ar_), 7.42 (d, *J* = 8.6 Hz, 2H, H_ar_) ppm.; ^13^C NMR (100 MHz, DMSO-*d_6_*): δ 55.1 (OCH_3_), 55.2 (OCH_3_), 70.0 (OCH_2_), 74.4 (OCH_2_), 113.5 (C_ar_), 113.9 (C_ar_), 117.1 (C_ar_), 121.4 (C_ar_), 124.1 (C_ar_), 128.0 (C_ar_), 128.7 (C_ar_), 129.5 (C_ar_), 129.7 (C_ar_), 130.0 (C_ar_), 146.6 (C_ar_O), 152.3 (C_ar_O), 159.0 (COCH_3_), 159.2 (COCH_3_), 167.5 (C = O) ppm; HRMS (ES+): exact mass calculated for C_23_H_22_O_6_Na: 417.1314. Found 417.1317.

*3-((4-methoxybenzyl)oxy)-2-methyl-4H-pyran-4-one (**5**)*

To a solution of 500 mg (3.96 mmol, 1 equiv.) of maltol in 5 mL of DMF was added 547 mg (3.96 mmol, 1 equiv.) of potassium carbonate and 0.6 mL (4.4 mmol, 1.1 equiv.) of 4-methoxybenzyl chloride. The reaction mixture was stirred at 60 °C for 5 h. The resulting solution was allowed to return to room temperature. The solvent was evaporated and water was added. The aqueous phase was washed with DCM (3 × 10 mL). The organic phases were washed with water (2 × 10 mL), dried with Na_2_SO_4_, filtrated and concentrated. The residue was purified by flash chromatography (AcOEt:Cyclohexane 7:3) to yield a yellow oil (650 mg, 65%). ^1^H NMR (400 MHz, CDCl_3_): δ 2.05 (s, 3H, CH_3_), 3.80 (s, 3H, OCH_3_), 5.10 (s, 2H, CH_2_), 6.36 (d, *J* = 5.64 Hz, 1H, CH-C = O), 6.85 (d, *J* = 8.6 Hz, 2H, H_ar_), 7.30 (d, *J* = 8.6 Hz, 2H, H_ar_), 7.58 (d, *J* = 5.64 Hz, 1H, CH-CO) ppm; ^13^C NMR (100 MHz, CDCl_3_): δ 14.8 (CH_3_), 55.2 (OCH_3_), 73.1 (OCH_2_), 113.7 (2C_ar_), 117.1 (C_ar_), 129.0 (C_ar_), 130.7 (2C_ar_), 143.5 (C-CO), 153.3 (CCH_3_), 159.7 (CH-CO), 159.8 (CH-C = O), 175.1 (C = O) ppm. HRMS (ES+): exact mass calculated for C_14_H_14_O_4_Na: 269.0790. Found 269.0791.

*N,N′-(piperazine-1,4-diylbis(propane-3,1-diyl))bis(2,3-bis((4-methoxybenzyl)oxy)benzamide) (**7**)*

To a solution, under argon, of 0.25 mL (1.2 mmol, 1 equiv.) 1,4-bis(3-aminopropyl)piperazine **3** in 100 mL of DCM was added 1 g (2.5 mmol, 2.1 equiv.) of **4**, 510 mg (2.65 mmol, 2.2 equiv.) of EDCI and 370 mg (2.41 mmol, 2 equiv.) of HOBt. The solution was stirred at 25 °C overnight. The resulting solution was concentrated under reduced pressure. The residue was purified by flash chromatography (DCM:MeOH 9:1) to yield a yellow oil (685 mg, 60%). ^1^H NMR (400 MHz, CDCl_3_): δ 1.52–1.69 (m, 4H, CH_2_), 1.69 (m, 8H, CH_2-pip_), 3.33 (q, J = 6.6 Hz, 4H, CH_2_), 2.25 (t, J = 7.2 Hz, 4H, CH_2_), 3.77 (s, 6H, OCH_3_), 3.82 (s, 6H, OCH_3_), 4.97 (s, 4H, OCH_2_), 5.06 (s, 4H, OCH_2_), 6.81 (d, J = 8.6 Hz, 4H, H_ar_), 6.93 (d, J = 8.6 Hz, 4H, H_ar_), 7.11–7.13 (m, 4H, H_ar_), 7.23 (d, J = 8.6 Hz, 4H, H_ar_), 7.38 (d, J = 8.6 Hz, 4H, H_ar_), 7.67 (dd, J = 6.0, 3.6 Hz, 2H, H_ar_), 8.16 (t, J = 8.0 Hz, 2H, NH) ppm. ^13^C NMR (100 MHz, CDCl_3_): δ 26.4 (CH_2_), 38.5 (CH_2_), 53.0 (CH_2-pip_), 55.4 (OCH_3_), 55.5 (OCH_3_), 56.4 (CH_2_), 71.2 (OCH_2_), 76.1 (OCH_2_), 114.0 (C_ar_), 114.2 (C_ar_), 116.9 (C_ar_), 123.2 (C_ar_), 124.4 (C_ar_), 128.0 (C_ar_), 128.7 (C_ar_), 128.8 (C_ar_), 129.6 (C_ar_), 130.7 (C_ar_), 146.8 (C_ar_O), 152.0 (C_ar_O), 159.8 (COCH_3_), 160.0 (COCH_3_), 165.3 (C = O) ppm; HRMS (ES+): exact mass calculated for C_56_H_65_N_4_O_10_: 953.4701. Found 953.4700.

*1,1′-(piperazine-1,4-diylbis(propane-3,1-diyl))bis(3-((4-methoxybenzyl)oxy)-2-methylpyridin-4(1H)-one) (**8**)*

0.6 mL (2.7 mmol, 1 equiv.) of 1,4-bis(3-aminopropyl)piperazine **3** was dissolved in 120 mL of a MeOH/H_2_O (2/1) solution. 2 g (8.1 mmol, 3 equiv.) of **5** and 250 mg (6.2 mmol, 2.3 equiv.) of sodium hydroxide were added. The reaction mixture was stirred under reflux for 3 days. The resulting solution was washed with DCM, and the organic layers were combined and dried over Na_2_SO_4_ and concentrated. The resulting mixture was treated with HCl 3M in MeOH, evaporated, and triturated with ether. The solid formed was washed with ether and DCM to yield a white solid (1.2 g, 70%). mp: 100 °C. ^1^H NMR (400 MHz, MeOH-d4): δ 2.02-2.14 (m, 4H, CH_2_), 2.49 (s, 6H, CH_3_), 2.83-3.08 (m, 12H, CH_2-pip_,CH_2_), 3.80 (s, 6H, OCH_3_), 4.40 (t, J = 7.5 Hz, 4H, CH_2_), 5.15 (s, 4H, OCH_2_), 6.92 (d, J = 8.6 Hz, 4H, H_ar_), 7.16 (d, J = 7.1 Hz, 2H, CH), 7.34 (d, J = 8.6 Hz, 4H, H_ar_), 8.35 (d, J = 7.1 Hz, 2H, CH) ppm; ^13^C NMR (100 MHz, MeOH-d_4_): δ 13.5 (CH_3_), 26.8 (CH_2_), 52.0 (CH_2_), 54.0 (CH_2_), 54.1 (CH_2-pip_), 55.8 (OCH_3_), 75.2 (OCH_2_), 114.9 (C_ar_), 115.4 (C_ar_), 129.7 (C_ar_), 132.0 (C_ar_), 142.5 (CCH_3_), 145.7 (CH), 149.0 (CO), 161.6 (COCH_3_), 169.4 (C = O) ppm; HRMS (ES+): exact mass calculated for C_38_H_49_N_4_O_6_: 657.3652. Found 657.3657.

*N,N′-(piperazine-1,4-diylbis(propane-3,1-diyl))bis(2,3-dihydroxybenzamide) (**1**)*

100 mg (0.15 mmol, 1 equiv.) of **7** was dissolved in 20 mL of a MeOH with 1 mL DCM. Using the H-cube, the resulting solution was put under H_2_ with 20% Pd(OH)_2_/C at 60 °C with a flow rate of 1 mL.min^−1^. The resulting solution was evaporated to yield a solid (50 mg, 90%). mp: 180 °C. ^1^H NMR (400 MHz, MeOH-d_4_): δ 1.92-1.99 (m, 4H, CH_2_), 2.92 (t, *J* = 7.5 Hz, 4H, CH_2_), 3.10-3.14 (m, 8H, CH_2-pip_), 3.49 (t, *J* = 6.7 Hz, 4H, CH_2_), 6.73 (t, *J* = 8.0 Hz, 2H, H_ar_), 6.94 (dd, *J* = 1.5, 8.0 Hz, 2H, H_ar_), 7.21 (dd, *J* = 1.5, 8.0 Hz, 2H, H_ar_) ppm; ^13^C NMR (100 MHz, MeOH-d_4_): δ 26.2 (CH_2_), 37.8 (CH_2_), 51.6 (CH_2-pip_), 55.8 (CH_2_), 116.9 (C_ar_), 118.9 (C_ar_), 119.7 (C_ar_), 119.7 (C_ar_), 121.8 (C_ar_), 147.4 (COH), 150.0 (COH), 171.8 (C = O) ppm; HRMS (ES+): exact mass calculated for C_24_H_33_N_4_O_6_: 473.2400. Found 473.2413. LCMS: tr 8.8 min (2% A/98% B), 190 nm: purity >96%.

*1,1′-(piperazine-1,4-diylbis(propane-3,1-diyl))bis(3-hydroxy-2-methylpyridin-4(1H)-one) (**2**)*

1 g (1.5 mmol, 1 equiv.) of **8** was dissolved in 100 mL of a MeOH. Using the H-cube, the resulting solution was put under H_2_ with 20% Pd(OH)_2_/C at 60 °C with a flow rate of 1 mL.min^−1^. The resulting solution was evaporated to yield a red solid (600 mg, 90%). mp: 260 °C. ^1^H NMR (400 MHz, MeOH-d_4_): δ 2.31-2.35 (m, 4H, CH_2_), 2.69 (s, 6H, CH_3_), 3.13-3.20 (m, 4H, CH_2_), 3.47–3.54 (m, 8H, CH_2-pip_), 4.52 (t, *J* = 7.7 Hz, 4H, CH_2_), 7.14 (d, *J* = 6.9 Hz, 2H, H_ar_), 8.24 (d, *J* = 6.9 Hz, 2H, H_ar_) ppm; ^13^C NMR (100 MHz, MeOH-d_4_): δ 12.9 (CH_3_), 26.0 (CH_2_), 50.4 (CH_2_), 54.2 (CH_2-pip_), 54.6 (CH_2_), 111.9 (C_ar_), 139.2 (CCH_3_), 143.4 (C_ar_), 145.3 (COH), 159.9 (C = O) ppm; HRMS (ES+): exact mass calculated for C_22_H_33_N_4_O_4_: 417.2502. Found 417.2500. LCMS: tr 0.9 min (98% A/2% B), 190 nm: purity >92%.

### 3.2. Physicochemical Studies

#### 3.2.1. Generalities

All commercial reagents used were of the highest purity (>99%) and were used without further purification. All stock solutions were used fresh. Because of the poor solubility of the chelators in aqueous media, all experiments were monitored in H_2_O/DMSO media (v/v = 50:50; x_DMSO_ = 0.2).

Ligand and metal solutions were prepared in the concentration range between 10^−4^ to 2 × 10^−3^ mol·L^−1^. The ionic strength was kept constant (I = 0.1) by addition of potassium chloride (Prolabo) of the highest purity (>99%). The titrating solutions of carbonate-free base KOH and hydrochloric acid 0.1M were prepared from standardized 1M solutions (Prolabo). All solutions were prepared with glass-distilled, deionized water degassed by argon saturation to remove all dissolved CO_2_ and with DMSO, spectrophotometric grade. Protometric titrations were carried out with an automatic titrator composed of a microprocessor burette (Metrohm Dosimat 665), a glass electrode (metrohm AG 9101) with an incorporated Ag/AgCl reference (filled with KCl 3 mol·L^−1^ and a pH meter (Metrohm 713) connected to a computer. The combined electrode has a very low alkaline error. The titration was fully automated. All measurements were performed within a thermoregulated cell at 25 °C under an argon stream to avoid the dissolution of carbon dioxide.

For a classical titration, a total of 120 to 150 points (volume of titrant, pH) was recorded. All equilibrium measurements were carried out in 4.00 mL sample volumes with magnetic stirring. The electrode slope was determined with two commercial buffers (pH 4 and pH 10) and checked by titration with an HCl solution at exactly 5.0 10^−3^ mol·L^−1^. No correction was necessary in the pH range of 2–12.5. The titrant, a carbonate-free KOH solution (Normadose, Merck, Darmstadt, Germany), was standardized against a 10^−2^ mol·L^−1^ potassium hydrogen phthalate solution by pH potentiometry. The ionic product of the H_2_O/DMSO was determined with the titration of hydrogen phthalate solutions, under our experimental conditions. A value of 16.0 was determined and used in the calculations. Consequently, the usable pH range is between 2 and 14.

UV-visible spectra were recorded after each base addition at (25 ± 0.1 °C). All spectra were recorded using a Shimadzu UV-2401-PC spectrophotometer equipped with a standard syringe shipper and a temperature-controlled TCC-240A cell holder. The experiments were monitored in the concentration range used for the pH titration; averages of 25 spectra were recorded in the pH range from 2 to 12.

#### 3.2.2. ESI-MS

All experiments were performed on a hybrid tandem quadrupole/time-of-flight (Q-TOF) instrument, equipped with a pneumatically assisted electrospray (Z-spray) ion source operated in positive or negative mode (SYNAPT G2si, Waters). The electrospray potential was set to 0.5 kV in positive and 2 kV in negative ion mode, and the extraction cone voltage was set to 40 V to obtain optimized mass spectra. The FeL solutions were prepared in similar conditions to those set out for the potentiometric study without ionic strength. The stoichiometries of the molecular associations were determined in accordance with the greater isotopic peak (corresponding to species with ^56^Fe), and further checked by simulating the different parts of the spectrum with the Isopro 3.0 software [42]. The differences between the experimental and calculated m/z values were less than or equal to 0.1 unit.

#### 3.2.3. Computation

The protonation constants of the ligand and the overall stability constants (β_mlh_) of the metal complexes were calculated from nonlinear least-square refinements of potentiometric titration with the general computation program PROTAF [43].

The global protonation constant β_m1h_

(1) $$\mathrm{mM}+\mathrm{lL}+\mathrm{hH}\;⇋\;M_{m}L_{l}H_{h},$$

(2) $$\beta_{\mathrm{m1h}}=\frac{\left[M_{m}L_{l}H_{h}\right]}{{\left[M\right]}^{m}{\left[L\right]}^{l}{\left[H\right]}^{h}},$$

in which m, l, and h are values in the general complex formula [M_m_L_l_H_h_]. M, L, and H correspond to the metal ion, the ligand, and the protons, respectively. For the sake of clarity, charges are omitted. The computer program HYSS was used to obtain the species distribution curves [44,45].

#### 3.2.4. pFe Determination

For ligands that have low water solubility, the classical methodology to determine pFe consists of determining the overall formation constant (logβ) values in a water/solvent mixture by varying the molar fraction of the used solvent (x_DMSO_). Then, the values are extrapolated in a pure H_2_O medium by taking into account a linear dependence approximation between logβ and the molar fraction of the solvent (x_DMSO_) [46]. The complex and ligand insolubility in a water/DMSO medium for x_DMSO_ < 0.2 renders such a methodology irrelevant to the pFe determination in the case of Fe-**1** and Fe-**2** systems. This problem can be overcome by performing pFe calculations with data collected in a H_2_O/DMSO medium (x_DMSO_ = 0.2) at a pH corresponding to the same [OH^−^]/[H_3_O^+^] ratio as that in pure water (pH 7.45) [47]. For x_DMSO_ equal to 0.2, the pH scale is extended from 0 to 16.0, and a neutral solution has a pH value of 8.05. Consequently, a pH of 7.45 in water corresponds to 8.44 in a H_2_O/DMSO medium (x_DMSO_ = 0.2). pFe calculations were monitored by using the HYSS software [44,45].

### 3.3. Biological Evaluation and Siderophore-Like Activity

#### 3.3.1. Bacterial Strains

The following strains were used for testing antibacterial susceptibility: *Pseudomonas aeruginosa* DSM 1117, and for siderophore-like activity, *P. aeruginosa* PAO1 as a wild type strain producing pyoverdine, and *P. aeruginosa* PAD07, a mutant strain, which does not produce pyoverdine or pyochelin, were used.

#### 3.3.2. Antibacterial Activity of 1 and 2

The different bacterial strains were incubated overnight at 35 °C in tryptic soy broth and streaked on tryptic soy agar. The inocula were then prepared according to the recommendations of CLSI (Clinical and Laboratory Standards Institute) and the Mueller-Hinton micro-dilution technique (MH, pH 7.3) using a concentration range of 0.25 to 512 mgL^−1^. These concentrations were obtained by dilutions from a stock solution of the tested product prepared in DMSO. Ciprofloxacin (Sigma Aldrich) in water was used as a control. The minimum inhibitory concentration (MIC) was determined visually and corresponds to the lowest concentration for which the wells are clear.

#### 3.3.3. Cytotoxicity

Compounds cytotoxicity was evaluated in a human hepatoma cell line (HepG2 from ECACC, MERCK) cultured in 75 cm^2^ sterile flasks in modified Dulbecco’s Modified Eagle’s Medium supplemented with 10% fetal bovine serum (Gibco) in a humidified atmosphere of 5% carbon dioxide at 37 °C. Concentrations of the compounds in the range 0–100 μM were added to cells. The vehicle (DMSO) was used as control. After a 48 h incubation period, cell viability was determined with the CellTiter-Glo^®^ Luminescent Cell Viability Assay (Promega), according to the manufacturer’s protocol. Cell viability was expressed as the percentage of Relative Light Units (RLU).

#### 3.3.4. Bacterial Growth under Restricted Iron Medium and Potential Siderophore-Like Activity

The medium used was the succinate minimum medium (SMM, pH = 7). This corresponds to a depleted iron medium and is obtained by mixing a solution of 6.0 g·L^−1^ of K_2_HPO_4_, a solution of 3.0 g.L^−1^ of KH_2_PO_4_, a solution containing 1.0 g.L^−1^ of NH_4_)_2_SO_4_, a solution of 0.2 g.L^−1^ of MgSO_4_.7H_2_O, and a solution of 4.0 g.L^−1^ of sodium succinate in PPI water (distilled water, sterile at pH = 7.0). This method simulates the conditions of development of bacteria in a depleted iron medium [40]. Our compounds were evaluated on this medium with or without addition of FeCl_3_ (Sigma Aldrich). For **1**, the FeCl_3_ concentration in wells was fixed at 67 μM, and those of **1** were ranged from 2.109 μM to 270 μM (molecular ratio from 0.03125 to 4). As for **2**, the FeCl_3_ concentration in wells was fixed at 65 μM, and those of **2** were ranged from 2.039 μM to 260 μM (molecular ratio from 0.03125 to 4). In a 96-well plate, 5 μL of each dilution of the tested products was distributed with 5 μL of sterile water or 5 μL of a sterile aqueous FeCl_3_ solution. Each well was then supplemented with 220 μL of SMM and 20 μL of a 0.5 McFarland bacterial suspension. The plates were incubated for 24 h at 37 °C. Absorbance (OD) was then measured using a Multiskan Ex reader (Thermo Labsystems, Issy les Moulineaux, France) at 600 nm to observe bacterial growth. The references were obtained by making wells containing the medium and the molecule, without inoculum, in order to measure the absorbance of our compounds at 600 nm. Controls, containing the bacterial strains in SMM, without siderophore and supplemented or not with Fe (III), were also carried out. The experiments were repeated three times. Statistical analyses were carried out using the Mann–Whitney test page of Vassarstats website. A *p* value below 0.05 was considered as significant [48].

## 4. Conclusions

We have synthesized two iron chelators **1** and **2** in four steps for **1** and three steps for **2** with an average yield of 40%. Physicochemical studies highlighted the great affinity of these two ligands towards Fe(III). For both, the pFe values calculated with the formation constants exceed 20. The ligand **1** (pFe = 28.04) has an iron-chelating ability higher than the ligand **2** (pFe = 24.38) and the Pvd (pFe = 27), the main endogenous siderophore of *P. aeruginosa*. In physiological pH, **1** forms 2:3 complexes with iron, whereas two species Fe_2_(**2**)_2_H_−1_ or Fe_2_(**2**)_2_H_−2_, corresponding to the deprotonation of Fe_2_(**2**)_2_ are observed for **2**. Both compounds have shown no antibacterial activities. The ligands **1** and **2** significantly increased the *P. aeruginosa* (PAD07, PAO1 strains) growth in iron-supplemented SMM. These results suggest that **1** and **2** could be internalized by the bacteria. The difference of bacterial growth for *P. aeruginosa* PAO1 compared to *P. aeruginosa* PAD07, in iron supplemented SMM, with the addition of **1** or **2**, could be due to a Pvd’s additional effect in iron transport. The greater growth effect of **1** compared to **2** could be partly explained in terms of an iron chelator property. According to these data, we used these iron piperazine-based chelators **1** and **2** to internalize toxic metal such as gallium. Unfortunately, the corresponding Ga(III)-**1** and **2** complexes showed no antibacterial activity against *P. aeruginosa* DSM 1117 strain. However, ligands **1** and **2** could be used to promote the transport of antibiotics in *Pseudomonas* species.

## Figures and Tables

**Table 1:** Protonation and acidity constants of **1** and **2**.

	1	2
pKa_1_	2.39 (4) ^1^	2.21 (5)
pKa_2_	3.66 (4)	2.82 (3)
pKa_3_	6.99 (6)	3.72 (8)
pKa_4_	8.67 (8)	7.17 (2)
pKa_5_	-	11.09 (1)
pKa_6_	-	11.79 (2)

^1^ The calculated uncertainties for logβ_m1h_ were determined on the basis of the standard deviation. Values in parentheses refer to estimated standard deviations for the last significant digit. I = 0.1 mol.L^−1^ (KCl); T = 298 K, H_2_O/DMSO media (v/v 50:50).

**Table 2:** Global formation constant of Fe(III)-**1** and Fe(III)-**2** complexes.

		Log *β* 1	Log *β* 2
Fe(LH)	log$\beta_{111}$	18.17 (6)^1^	-
Fe_2_(LH)_2_	log$\beta_{222}$		67.25 (3)
Fe_2_L_2_	log$\beta_{220}$	31.87 (6)	61.73 (4)
Fe_2_L(LH_−1_) or Fe_2_L_2_(OH)	log$\beta_{22-1}$	27.0 (5)	58.58 (4)
Fe_2_(LH_−1_)_2_ or Fe_2_L_2_(OH)_2_	log$\beta_{22-2}$	23.4 (1)	54.68 (5)
Fe_2_(LH_−2_)(LH_−1_) or Fe_2_L_2_(OH)_3_	log$\beta_{22-3}$	18.8 (1)	47.45 (6)
Fe_2_L_3_	log$\beta_{230}$		79.43 (1)
Fe_2_(LH_−1_)_3_	log$\beta_{23-3}$	27.92 (3)	
Fe_2_(LH_−1_)_2_(LH_−2_)	log$\beta_{23-4}$	18.8 (5)	
Fe_2_(LH_−1_)(LH_−2_)_2_	log$\beta_{23-5}$	10.2 (6)	
Fe_2_(LH_−2_)_3_	log$\beta_{23-6}$	0.6 (1)	

^1^ Values in parentheses refer to estimated standard deviations.

**Table 3:** Antipseudomonal activities and cytotoxicity of **1** and **2**.

	Activity	Cytotoxicity
Compound	MIC ^1^ (mg·L^−1^)	IC_50_^2^ (mmol·L^−1^)
**1**	>512 (MH) ^3^ >512 (SMM) ^4^	>100
**2**	>512 (MH) >512 (SMM)	>100
Ciprofloxacin	0.5 (MH) 0.5 (SMM)	-

^1^ MIC: Minimum Inhibitory Concentration on *P. aeruginosa* DSM 1117 strains; ^2^ IC_50_: half maximal Inhibitory Concentration on Hep-G2 cell line; ^3^ MH: Mueller-Hinton Medium; ^4^ SMM: Succinate Minimum Medium.

**Table 4:** Antipseudomonal activities and cytotoxicity of **Ga(III)-1** and **Ga(III)-2.**

	Activity	Cytotoxicity
Compound	MIC ^1^ (mg.L^−1^)	IC_50_^2^ (mmol.L^−1^)
**Ga(III)-1**	>512 (MH) ^3^ >512 (SMM) ^4^	>100
**Ga(III)-2**	>512 (MH) >512 (SMM)	>100

^1^ MIC: Minimum Inhibitory Concentration on *P. aeruginosa* DSM 1117 strains; ^2^ IC_50_: half maximal Inhibitory Concentration on Hep-G2 cell line; ^3^ MH: Mueller-Hinton Medium; ^4^ SMM: Succinate Minimum Medium.

## Supplementary Materials

The following are available online at https://www.mdpi.com/1424-8247/12/4/160/s1.

## References

[1] Santé Publique France (2018). Enquête Nationale de Prévalence des Infections Nosocomiales et Des Traitements Anti-Infectieux en Établissements de Santé, Mai-Juin 2017.

[2] Schweizer H.P. (2012). Mechanisms of antibiotic resistance in *Burkholderia pseudomallei*: Implications for treatment of melioidosis. Future Microbiol..

[3] Inglis T.J.J., Rolim D.B., Sousa A.D.Q. (2006). Melioidosis in the Americas. Am. J. Trop. Med. Hyg..

[4] Benoit T.J., Elrod M.G., Walke H.T., Inglis T.J.J., Bower W.A., Gee J.E., Doker T.J., Hoffmaster A.R., Rolim D.B., Blaney D.D. (2015). A review of melioidosis cases in the Americas. Am. J. Trop. Med. Hyg..

[5] Limmathurotsakul D., Golding N., Dance D.A.B., Messina J.P., Pigott D.M., Moyes C.L., Rolim D.B., Bertherat E., Day N.P.J., Peacock S.J. (2016). Predicted global distribution of *Burkholderia pseudomallei* and burden of melioidosis. Nat. Microbiol..

[6] Mislin G.L.A., Schalk I.J. (2014). Siderophore-dependent iron uptake systems as gates for antibiotic Trojan horse strategies against *Pseudomonas aeruginosa*. Metallomics.

[7] Pham T., Loupias P., Dassonville-Klimpt A., Sonnet P. (2019). Drug delivery systems designed to overcome antimicrobial resistance. Med. Res. Rev..

[8] Neilands J.B. (1981). Iron absorption and transport in microorganisms. Annu. Rev. Nutr..

[9] Ratledge C. (2007). Iron metabolism and infection. Food Nutr. Bull..

[10] Pollack J.R., Neilands J.B. (1970). Enterobactin, an iron transport compound from *Salmonella Typhimurium*. Biochem. Biophys. Res. Commun..

[11] Neilands J.B. (1995). Siderophores: Structure and function of microbial iron transport compounds. J. Biol. Chem..

[12] Braun V., Killmann H. (1999). Bacterial solutions to the iron-supply problem. Trends Biochem. Sci..

[13] Hider R.C., Kong X. (2010). Chemistry and biology of siderophores. Nat. Prod. Rep..

[14] Nader M., Journet L., Meksem A., Guillon L., Schalk I.J. (2011). Mechanism of ferripyoverdine uptake by *Pseudomonas aeruginosa* outer membrane transporter FpvA: No diffusion channel formed at any time during ferrisiderophore uptake. Biochemistry.

[15] Valappil S.P., Yiu H.H.P., Bouffier L., Hope C.K., Evans G., Claridge J.B., Higham S.M., Rosseinsky M.J. (2013). Effect of novel antibacterial gallium-carboxymethyl cellulose on *Pseudomonas aeruginosa*. Dalton Trans..

[16] Page M.G.P., Dantier C., Desarbre E. (2010). In vitro properties of BAL30072, a novel siderophore sulfactam with activity against multiresistant Gram-Negative bacilli. Antimicrob. Agents Chemother..

[17] Schell M.A., Zhao P., Wells L. (2011). Outer membrane proteome of *Burkholderia pseudomallei* and *Burkholderia mallei* from diverse growth conditions. J. Proteome Res..

[18] Mima T., Kvitko B.H., Rholl D.A., Page M.G.P., Desarbre E., Schweizer H.P. (2011). In vitro activity of BAL30072 against *Burkholderia pseudomallei*. Int. J. Antimicrob. Agents.

[19] Luscher A., Moynié L., Auguste P.S., Bumann D., Mazza L., Pletzer D., Naismith J.H., Köhler T. (2018). TonB-dependent receptor repertoire of *Pseudomonas aeruginosa* for uptake of siderophore-drug conjugates. Antimicrob. Agents Chemother..

[20] Portsmouth S., van Veenhuyzen D., Echols R., Machida M., Ferreira J.C.A., Ariyasu M., Tenke P., Nagata T.D. (2018). Cefiderocol versus imipenem-cilastatin for the treatment of complicated urinary tract infections caused by Gram-negative uropathogens: A phase 2, randomised, double-blind, non-inferiority trial. Lancet Infect. Dis..

[21] Ito A., Sato T., Ota M., Takemura M., Nishikawa T., Toba S., Kohira N., Miyagawa S., Ishibashi N., Matsumoto S. (2018). In vitro antibacterial properties of cefiderocol, a novel siderophore cephalosporin, against Gram-Negative bacteria. Antimicrob. Agents Chemother..

[22] Fardeau S., Dassonville-Klimpt A., Audic N., Sasaki A., Pillon M., Baudrin E., Mullié C., Sonnet P. (2014). Synthesis and antibacterial activity of catecholate—Ciprofloxacin conjugates. Bioorg. Med. Chem..

[23] Antonietti V., Boudesocque S., Dupont L., Farvacques N., Cézard C., Da Nascimento S., Raimbert J.F., Socrier L., Robin T.J., Morandat S. (2017). Synthesis, iron(III) complexation properties, molecular dynamics simulations and *P. aeruginosa* siderophore-like activity of two pyoverdine analogs. Eur. J. Med. Chem..

[24] Rathi A.K., Syed R., Shin H.S., Patel R.V. (2016). Piperazine derivatives for therapeutic use: A patent review (2010-present). Expert Opin. Ther. Pat..

[25] Magotra A. (2018). Physicochemical, pharmacokinetic, efficacy and toxicity profiling of a potential nitrofuranyl methyl piperazine derivative IIIM-MCD-211 for oral tuberculosis therapy via in-silico–in-vitro–in-vivo approach. Pulm. Pharmacol. Ther..

[26] Kharb R., Bansal K., Sharma A.K. (2012). A valuable insight into recent advances on antimicrobial activity of piperazine derivatives. Pharma Chem..

[27] Schiller D., Fung H. (2007). Posaconazole: An extended-spectrum triazole antifungal agent. Clin. Ther..

[28] Kern W.V., Steinke P., Schumacher A., Schuster S. (2006). Effect of 1-(1-naphthylmethyl)-piperazine, a novel putative efflux pump inhibitor, on antimicrobial drug susceptibility in clinical isolates of *Escherichia coli*. J. Antimicrob. Chemother..

[29] Leite A., Silva A.M.G., Nunes A., Andrade M., Sousa C., Cunha-Silva L., Gameiro P., de Castro B., Rangel M. (2011). Novel tetradentate chelators derived from 3-hydroxy-4-pyridinone units: Synthesis, characterization and aqueous solution properties. Tetrahedron.

[30] Gao J., Xing F., Bai Y., Zhu S. (2014). Synthesis, spectroscopy, and binding constants of ketocatechol-containing iminodiacetic acid and its Fe(III), Cu(II), and Zn(II) complexes and reaction of Cu(II) complex with H_2_O_2_ in aqueous solution. Dalton Trans..

[31] Allam A., Dechamps-Olivier I., Behr J.B., Dupont L., Plantier-Royon R. (2011). Thermodynamic, spectroscopic studies and catechol oxidase activity of copper (II) complexes with amphiphilic *d*-galacturonic acid derived ligands. Inorg. Chim. Acta.

[32] Nurchi V.M., Crisponi G., Pivetta T., Donatoni M., Remelli M. (2008). Potentiometric, spectrophotometric and calorimetric study on iron(III) and copper(II) complexes with 1,2-dimethyl-3-hydroxy-4-pyridinone. J. Inorg. Biochem..

[33] Charkoudian L.K., Franz K.J. (2006). Fe(III)-coordination properties of neuromelanin components: 5,6-dihydroxyindole and 5,6-dihydroxyindole-2-carboxylic acid. Inorg. Chem..

[34] Elhabiri M., Carrër C., Marmolle F., Traboulsi H. (2007). Complexation of iron(III) by catecholate-type polyphenols. Inorg. Chim. Acta.

[35] Miethke M., Marahiel M.A. (2007). Siderophore-based iron acquisition and pathogen control. Microbiol. Mol. Biol. Rev..

[36] Cézard C., Farvacques N., Sonnet P. (2015). Chemistry and biology of pyoverdines, *Pseudomonas* primary siderophores. Curr. Med. Chem..

[37] Albrecht-Gary A.M., Blanc S., Rochel N., Ocaktan A.Z., Abdallah M.A. (1994). Bacterial iron transport: Coordination properties of pyoverdin PaA, a peptidic siderophore of *Pseudomonas aeruginosa*. Inorg. Chem..

[38] Clinical and Laboratory Standards Institute (2015). Methods for Dilution Antimicrobial Susceptibility Tests for Bacteria That Grow Aerobically: M07-A10.

[39] Takase H., Nitanai H., Hoshino K., Otani T. (2000). Impact of siderophore production on *Pseudomonas aeruginosa* infections in immunosuppressed mice. Infect. Immun..

[40] Meyer J.M., Abdallah M.A. (1978). The fluorescent pigment of *Pseudomonas fluorescens*: Biosynthesis, purification and physicochemical properties. J. Gen. Microbiol..

[41] Bergeron R.J., Bharti N., Singh S., McManis J.S., Wiegand J., Green L.G. (2009). Vibriobactin antibodies: A vaccine strategy. J. Med. Chem..

[42] Senko M. (2017). Isopro 3.0 MS/MS Software.

[43] Fournaise R., Petitfaux C. (1987). Etude de la formation des complexes en solution aqueuse-III Nouvelle méthode d’affinement des constantes de stabilité des complexes et des autres paramètres des titrages protométriques. Talanta.

[44] Gans P., Sabatini A., Vacca A. (1996). Investigation of equilibria in solution. Determination of equilibrium constants with the HYPERQUAD suite of programs. Talanta.

[45] Alderighi L., Gans P., Ienco A., Peters D., Sabatini A., Vacca A. (1999). Hyperquad simulation and speciation (HySS): A utility program for the investigation of equilibria involving soluble and partially soluble species. Coord. Chem. Rev..

[46] Steinhauser S., Heinz U., Bartholomä M., Weyhermüller T., Nick H., Hegetschweiler K. (2004). Complex formation of ICL670 and related ligands with Fe(III) and Fe(II). Eur. J. Inorg. Chem..

[47] Rouge P., Dassonville-Klimpt A., Cézard C., Boudesocque S., Ourouda R., Amant C., Gaboriau F., Forfar I., Guillon J., Guillon E. (2012). Synthesis, physicochemical studies, molecular dynamics simulations, and metal-ion-dependent antiproliferative and antiangiogenic properties of cone ICL670-substituted calix [4] arenes. ChemPlusChem.

[48] Lowry R. Mann-Whitney Test. http://vassarstats.net.

