# Supplementary material for: Study of Iron Piperazine-Based Chelators as Potential Siderophore Mimetics
Source: Pharmaceuticals (Basel). 2019 Oct 23;12(4):160. doi: 10.3390/ph12040160 (PMC6958365; doi:10.3390/ph12040160)
Supplement: Supplementary file 1 [file pharmaceuticals-12-00160-s001.pdf]

Article

Study of iron piperazine-based chelators as potential siderophore mimetics

Pauline Loupias[1], Isabelle Dechamps[2], Laurent Dupont[2], Pierre Vanlemmens[1], Catherine Mullié[1], Nicolas Taudon[3], Anne Bouchut[1], Alexandra Dassonville-Klimpt[1], and Pascal Sonnet[1,*]

[1] AGIR, EA 4294, UFR de Pharmacie, Université de Picardie Jules Verne, 1 rue des Louvels, 80037 Amiens;
[2] ICMR, UMR 7312, 3 Avenue du Maréchal Juin, 51100 Reims;
[3] Unité de Toxicologie Analytique, Institut de Recherche Biomédicales des Armées, 91223 Brétigny-sur-Orge.
* Correspondence: pascal.sonnet@u-picardie.fr

NMR, HRMS, HPLC spectra of compounds **1 and 2** p.2-7

NMR, HRMS spectra of compounds **4-8** p.8-17

General procedure for the synthesis of gallium complexes **Ga(III)-**1 and **Ga(III)-2** p.18

N,N'-(piperazine-1,4-diylbis(propane-3,1-diyl))bis(2,3-dihydroxybenzamide) (1)

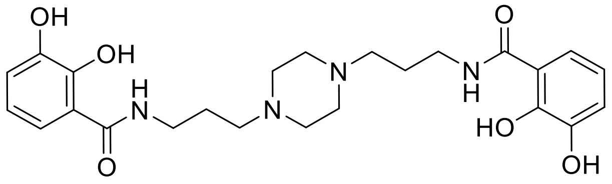

^{1}H NMR

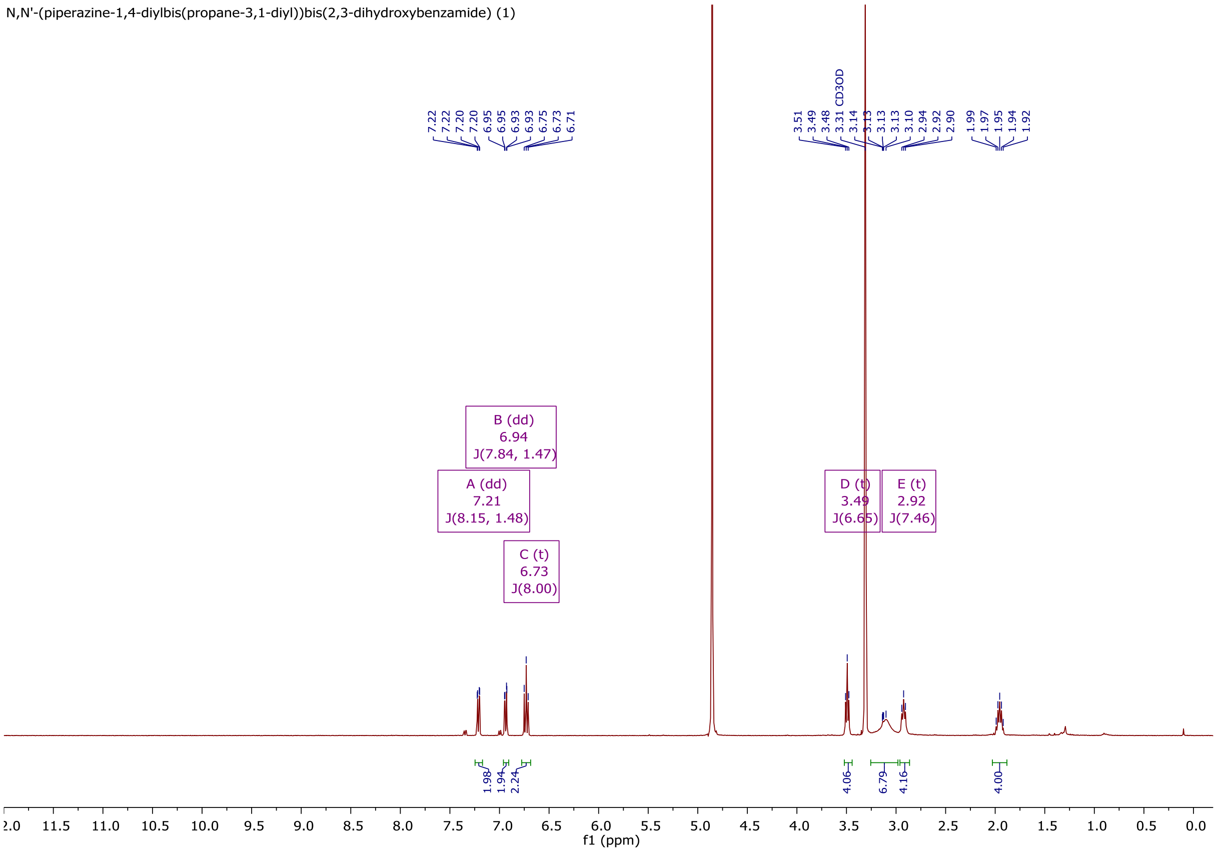

^{13}C NMR

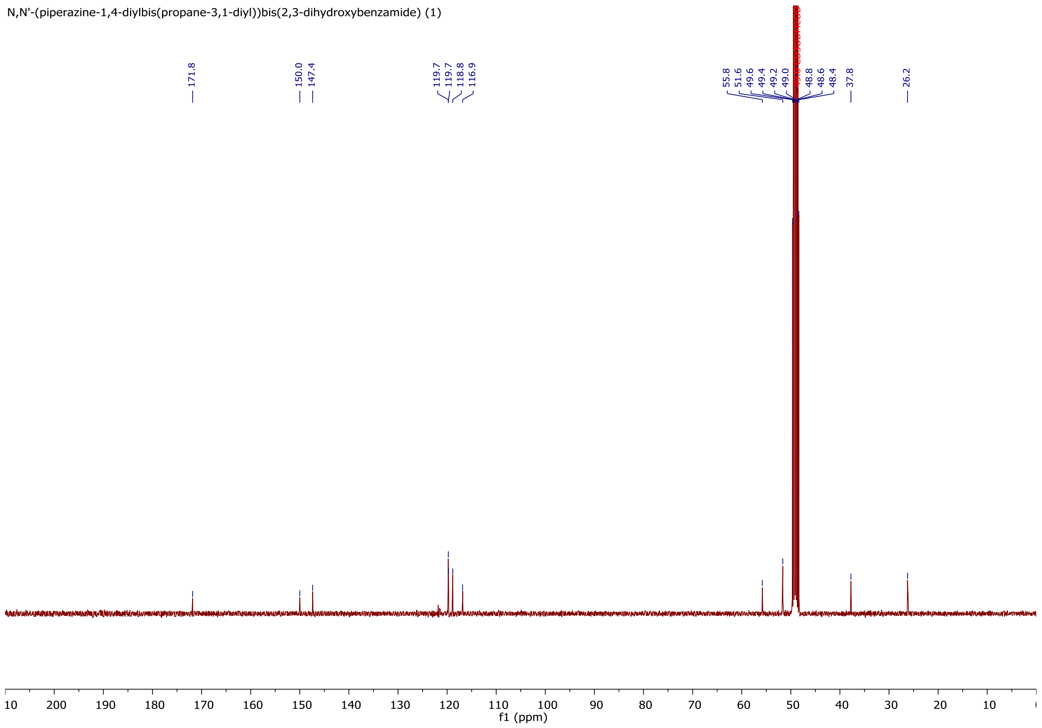

HRMS

Single Mass Analysis
Tolerance = 5.0 PPM / DBE: min = -1.5, max = 50.0
Element prediction: Off
Number of isotope peaks used for i-FIT = 3

Monoisotopic Mass, Even Electron Ions
80 formula(e) evaluated with 1 results within limits (all results (up to 1000) for each mass)
Elements Used:
C: 0-25 H: 0-35 N: 1-5 O: 1-10
PL-1-53 24 (0.205) Cm (24:30)
1: TOF MS ES+

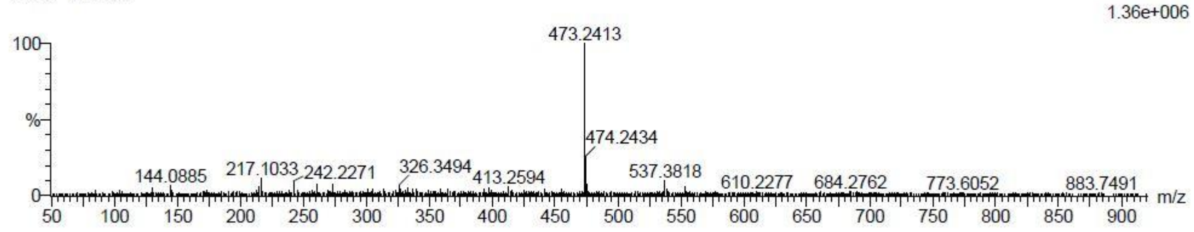

Minimum:				-1.5				
Maximum:		5.0	5.0	50.0				
Mass	Calc. Mass	mDa	PPM	DBE	i-FIT	Norm	Conf(%)	Formula
473.2413	473.2400	1.3	2.7	10.5	34.6	n/a	n/a	C24 H33 N4 O6

HPLC

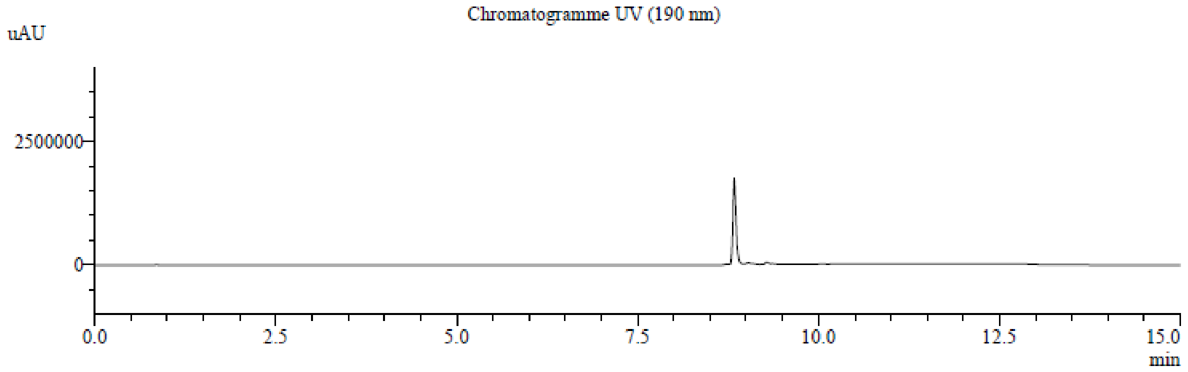

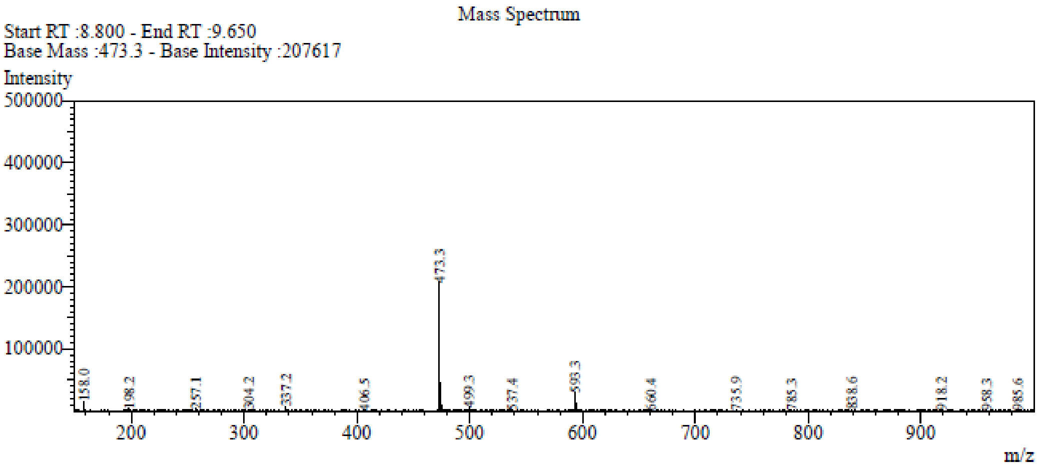

Peak Table

PDA Ch1 254nm

Peak#	Ret. Time	Area	Area%
1	8.834	5452712	96.104
2	9.028	116338	2.050
3	9.281	104724	1.846
Total		5673774	100.000

1,1′-(piperazine-1,4-diylbis(propane-3,1-diyl))bis(3-hydroxy-2-methylpyridin-4(1H)-one) (**2**)

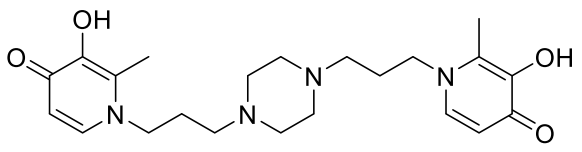

^{1}H NMR

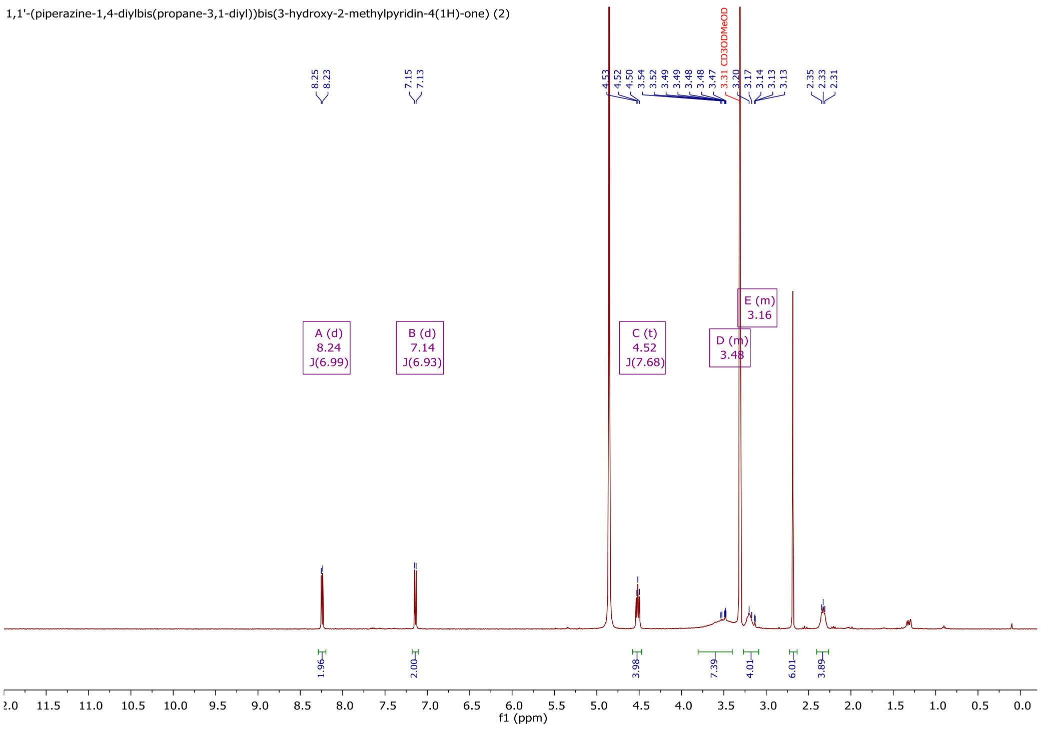

^{13}C NMR

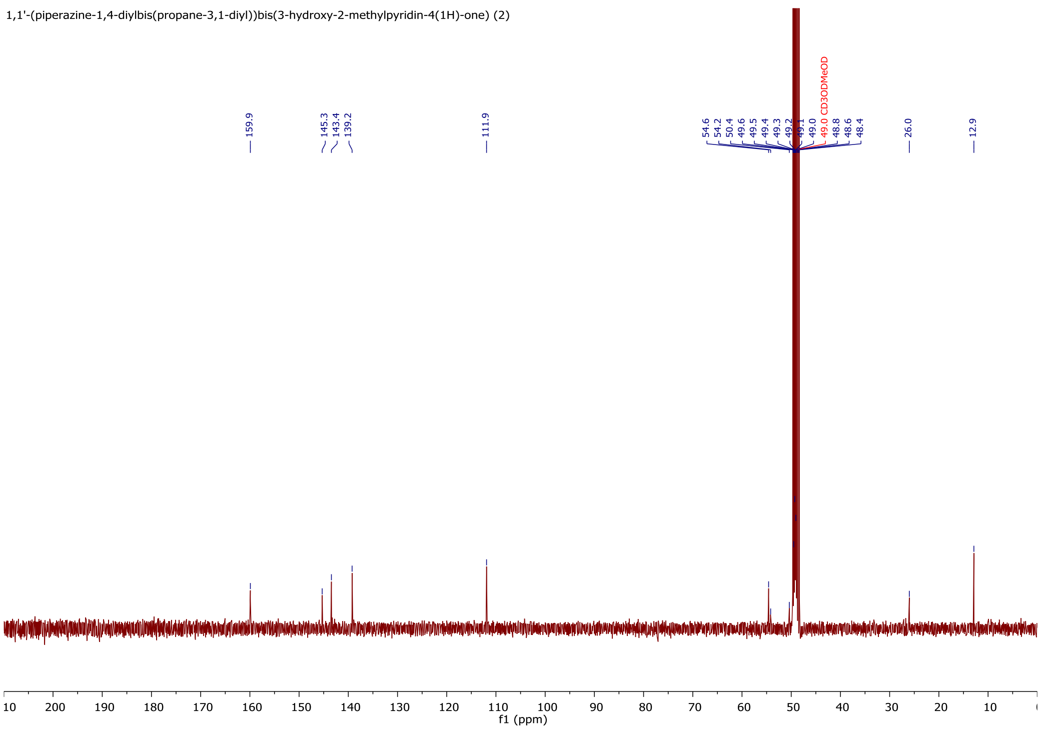

HRMS

Single Mass Analysis
Tolerance = 1.0 PPM / DBE: min = -1.5, max = 150.0
Element prediction: Off
Number of isotope peaks used for i-FIT = 4

Monoisotopic Mass, Even Electron Ions
1066 formula(e) evaluated with 1 results within limits (up to 50 closest results for each mass)
Elements Used:
C: 0-30 H: 0-40 N: 0-20 O: 0-20

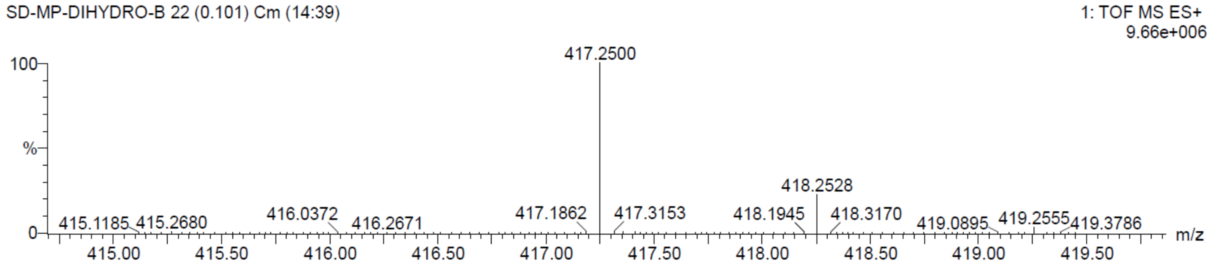

Minimum:				-1.5				
Maximum:		2.0	1.0	150.0				
Mass	Calc. Mass	mDa	PPM	DBE	i-FIT	Norm	Conf(%)	Formula
417.2500	417.2502	-0.2	-0.5	8.5	869.0	n/a	n/a	C22 H33 N4 O4

HPLC

Chromatogramme UV (190 nm)

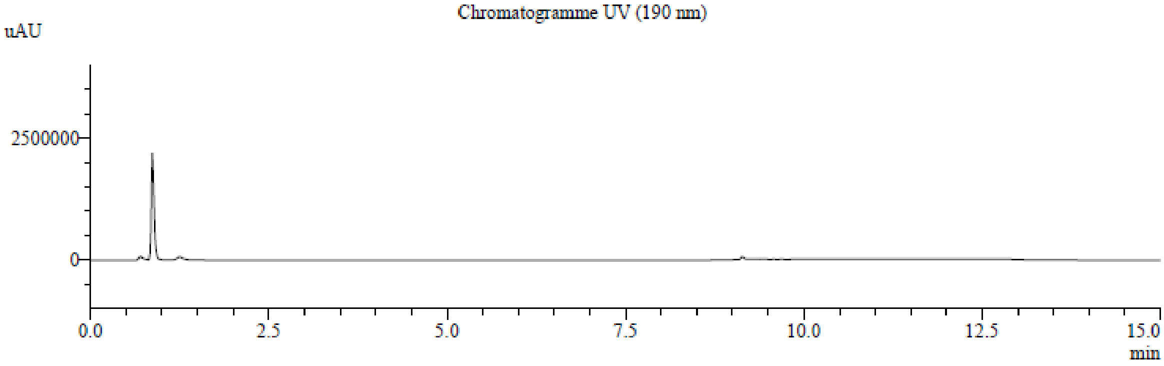

Mass Spectrum

Start RT :0.900 - End RT :1.350
Base Mass :132.6 - Base Intensity :26404

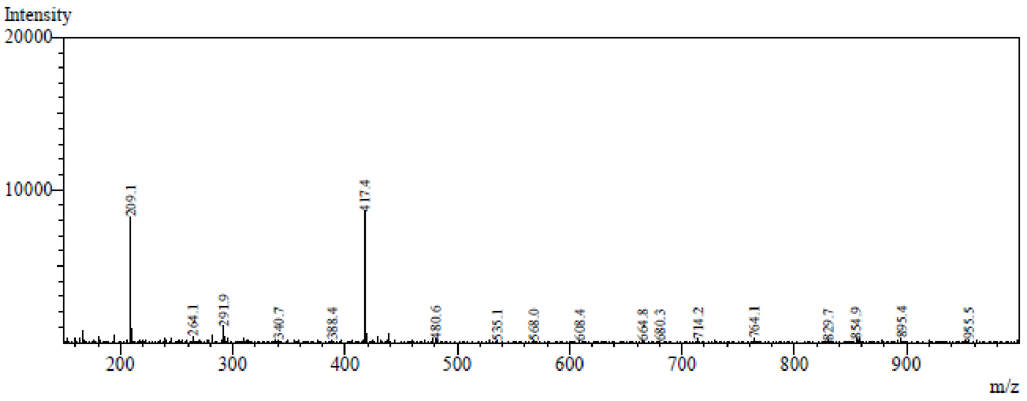

Peak Table

PDA Ch1 254nm

Peak#	Ret. Time	Area	Area%
1	0.699	263731	3.898
2	0.867	6206275	91.737
3	1.252	295270	4.364
Total		6765277	100.000

*2,3-bis((4-methoxybenzyl)oxy)benzoic acid (**4**)*

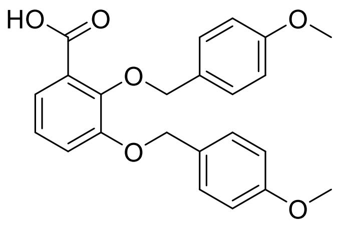

^{1}H NMR

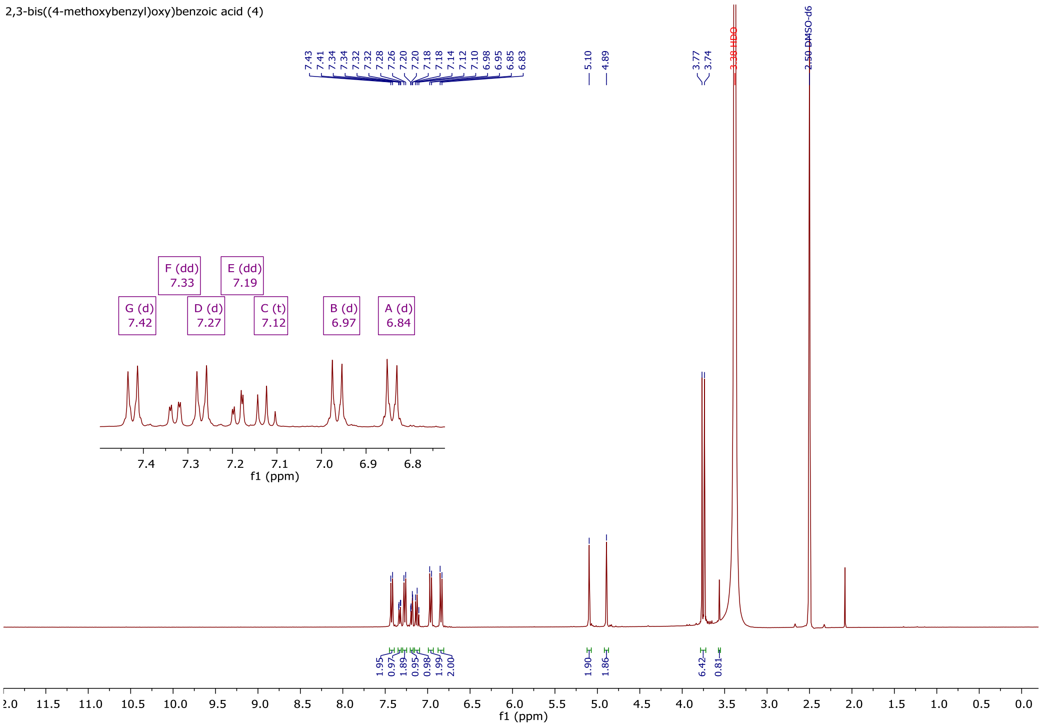

^{13}C NMR

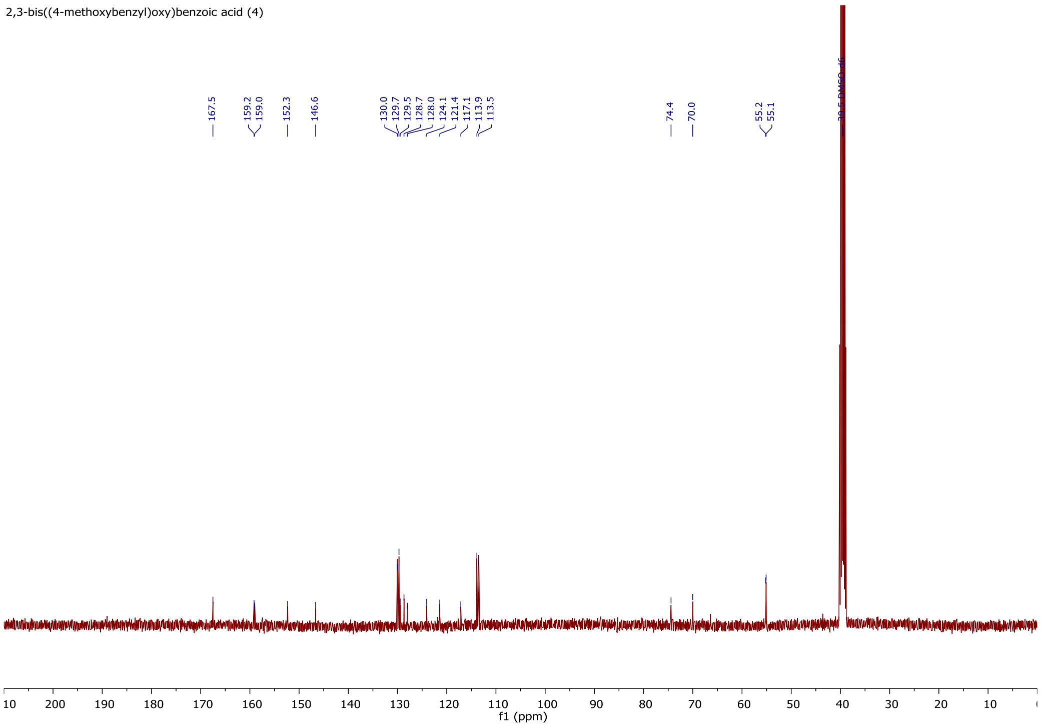

HRMS

Elemental Composition Report **Page 1**

Single Mass Analysis
Tolerance = 5.0 PPM / DBE: min = -1.5, max = 50.0
Element prediction: Off
Number of isotope peaks used for i-FIT = 3

Monoisotopic Mass, Even Electron Ions
26 formula(e) evaluated with 1 results within limits (all results (up to 1000) for each mass)
Elements Used:
C: 0-25 H: 0-25 O: 1-10 Na: 0-1
PL1-06 19 (0.159)
1: TOF MS ES+

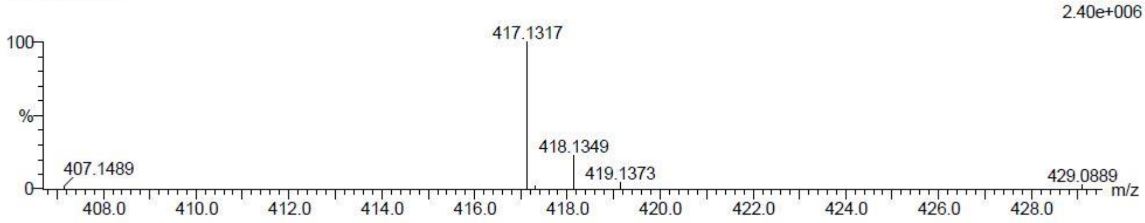

Mass	Calc. Mass	mDa	PPM	DBE	i-FIT	Norm	Conf(%)	Formula
Minimum:				-1.5				
Maximum:		5.0	5.0	50.0				
417.1317	417.1314	0.3	0.7	12.5	47.2	n/a	n/a	C23 H22 O6 Na

*3-((4-methoxybenzyl)oxy)-2-methyl-4H-pyran-4-one (**5**)*

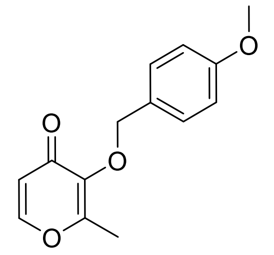

^{1}H NMR

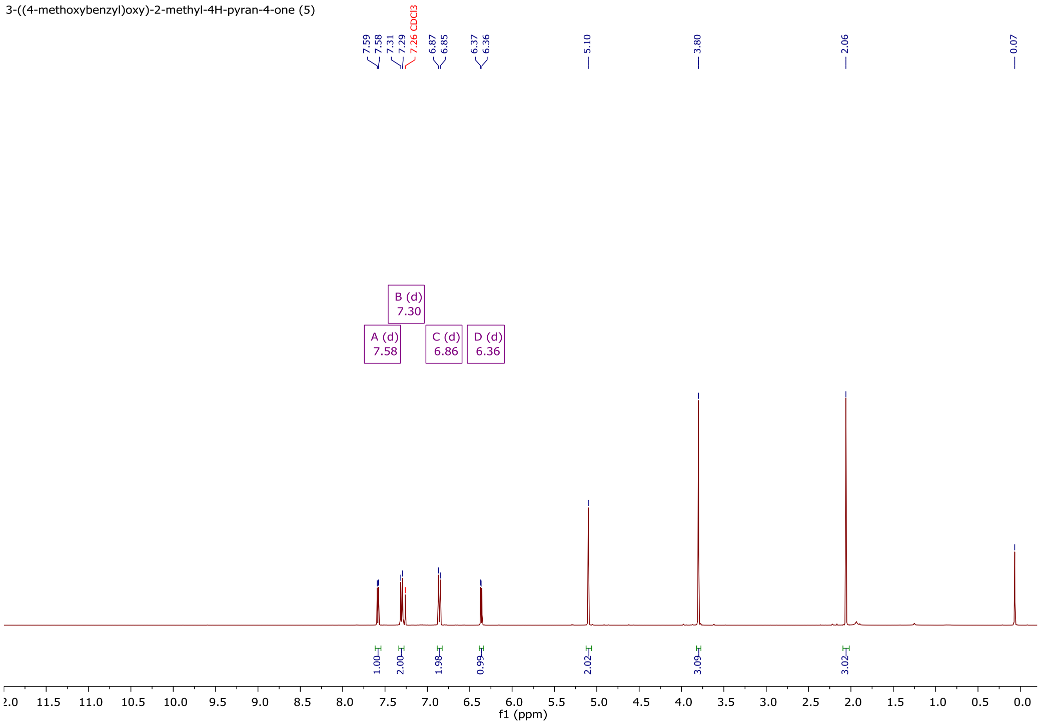

^{13}C NMR

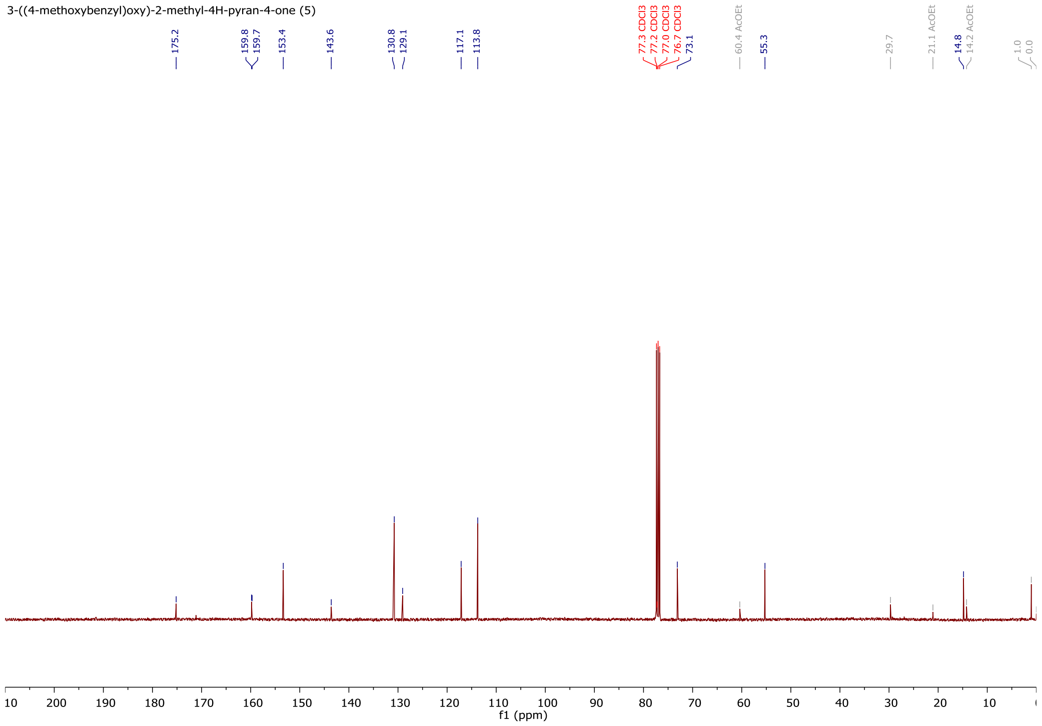

HRMS

Single Mass Analysis
Tolerance = 2.0 PPM / DBE: min = -1.5, max = 150.0
Element prediction: Off
Number of isotope peaks used for i-FIT = 4

Monoisotopic Mass, Even Electron Ions
95 formula(e) evaluated with 1 results within limits (up to 50 closest results for each mass)
Elements Used:
C: 0-40 H: 0-60 O: 0-10 Na: 0-2

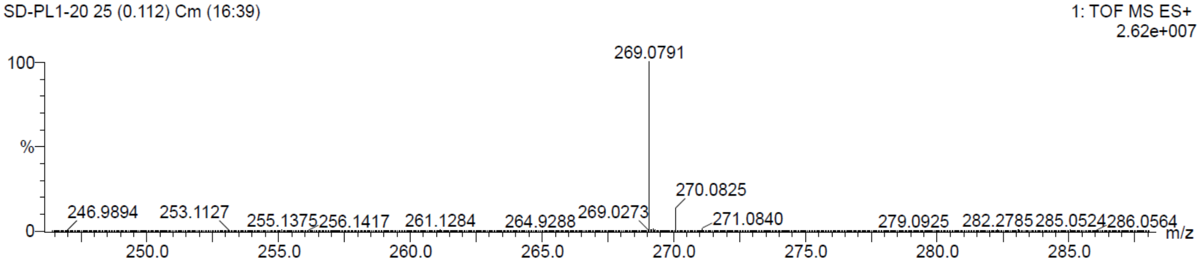

Minimum:				-1.5				
Maximum:		2.0	2.0	150.0				
Mass	Calc. Mass	mDa	PPM	DBE	i-FIT	Norm	Conf(%)	Formula
269.0791	269.0790	0.1	0.4	7.5	1325.0	n/a	n/a	C14 H14 O4 Na

*4-methoxybenzyl 2,3-bis((4-methoxybenzyl)oxy)benzoate (**6**)*

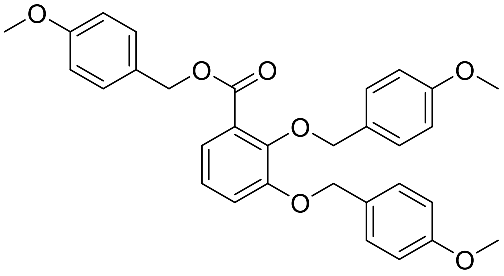

^{1}H NMR

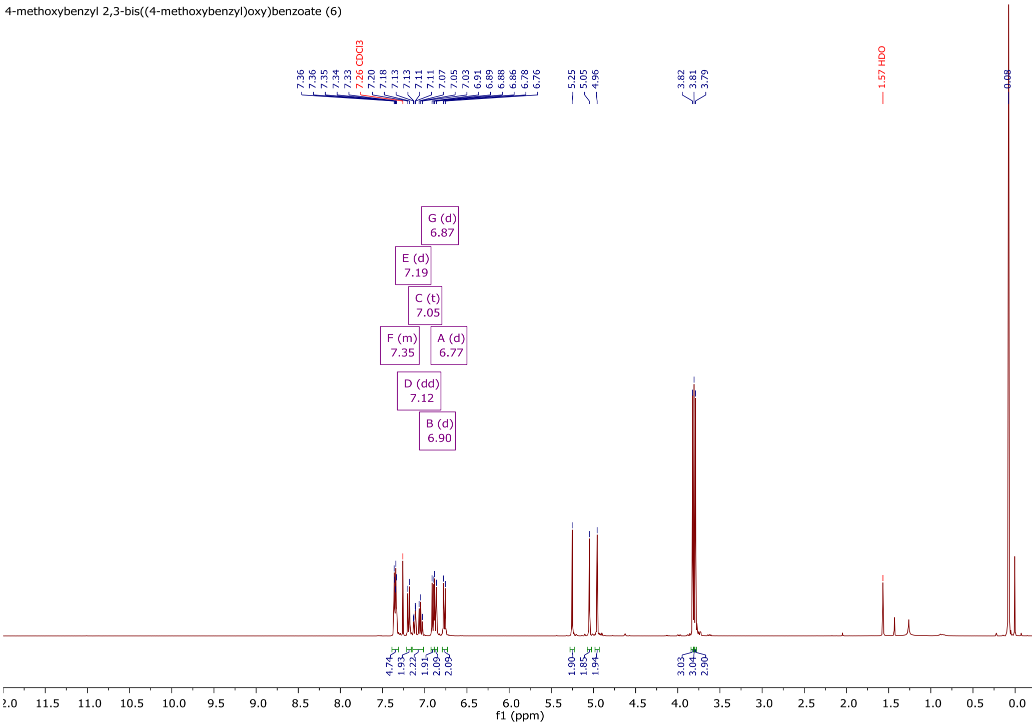

^{13}C NMR

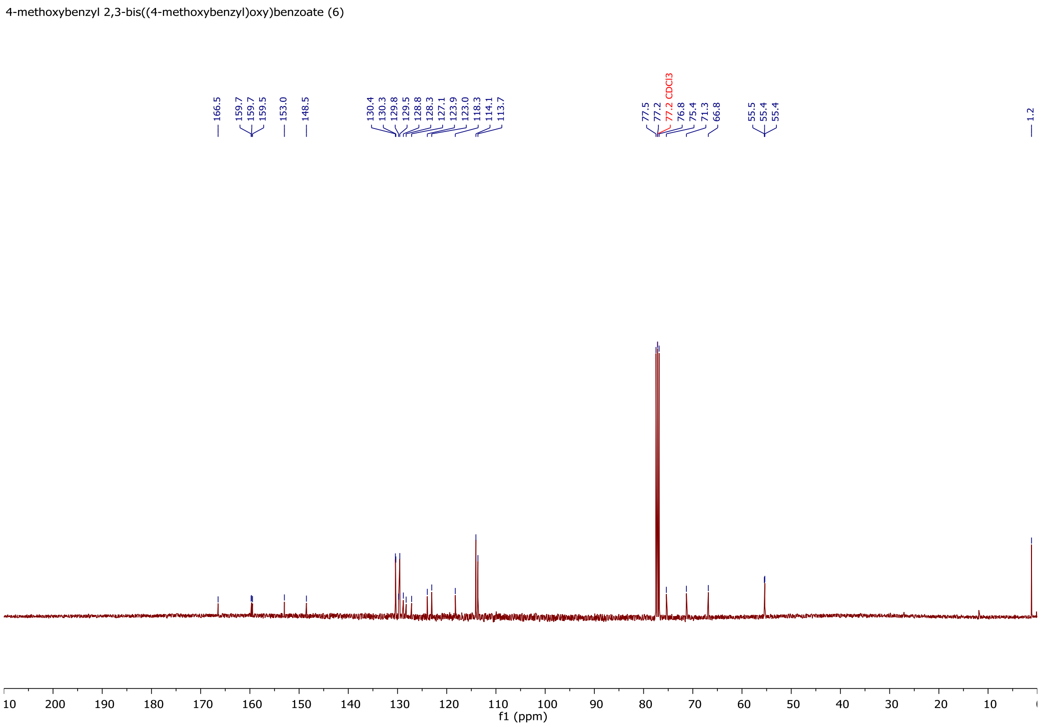

HRMS

Single Mass Analysis
Tolerance = 2.0 PPM / DBE: min = -1.5, max = 150.0
Element prediction: Off
Number of isotope peaks used for i-FIT = 4

Monoisotopic Mass, Even Electron Ions
155 formula(e) evaluated with 1 results within limits (up to 50 closest results for each mass)
Elements Used:
C: 0-40 H: 0-60 O: 0-10 Na: 0-2

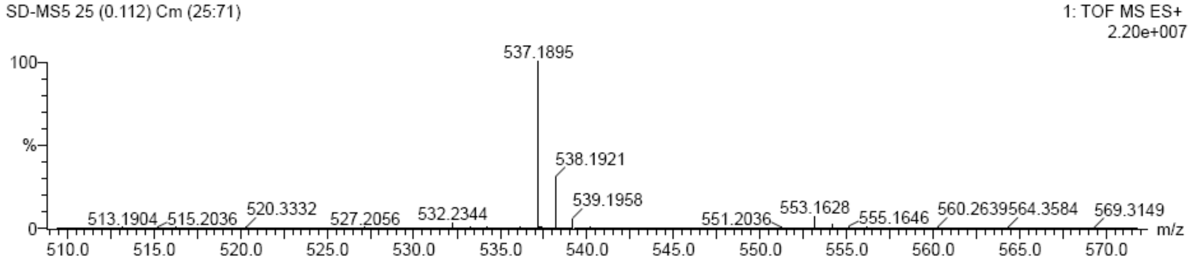

Minimum:				-1.5				
Maximum:		2.0	2.0	150.0				
Mass	Calc. Mass	mDa	PPM	DBE	i-FIT	Norm	Conf(%)	Formula
537.1895	537.1889	0.6	1.1	16.5	1360.7	n/a	n/a	C31 H30 O7 Na

N,N′-(piperazine-1,4-diylbis(propane-3,1-diyl))bis(2,3-bis((4-methoxybenzyl)oxy)benzamide) (7)

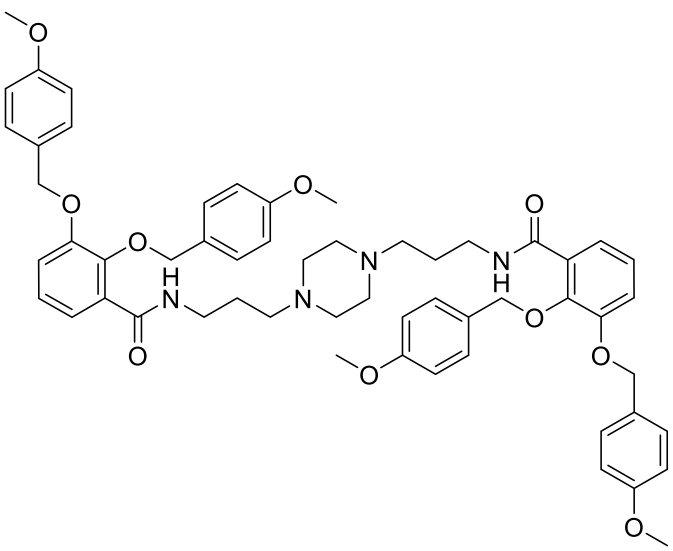

<u>¹H NMR</u>

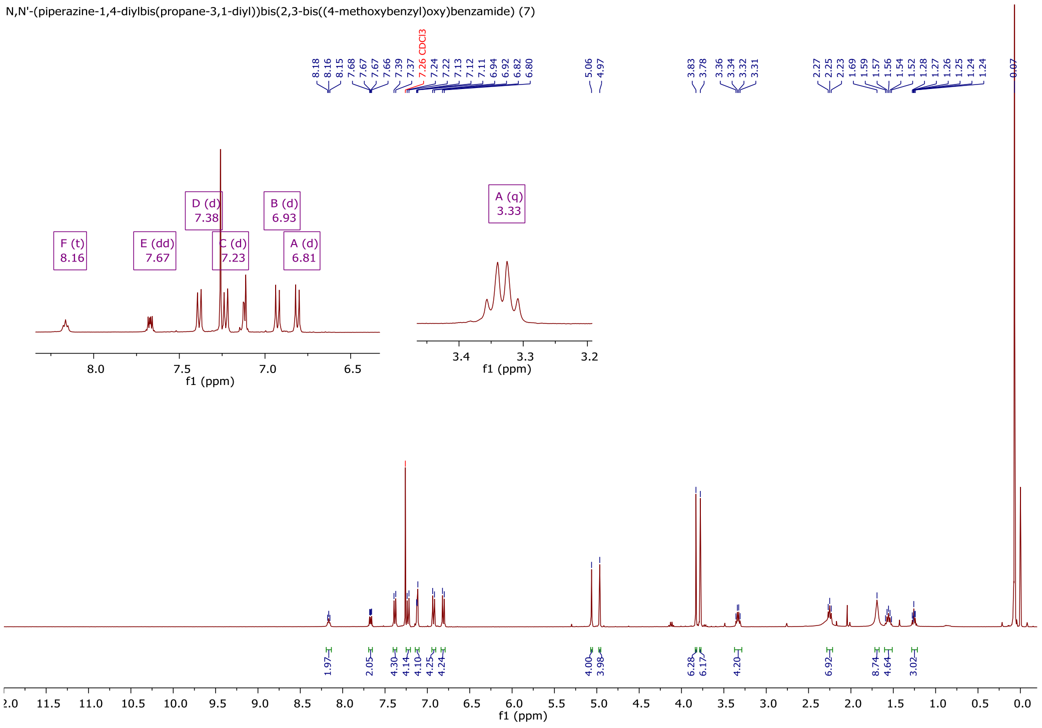

^{13}C NMR

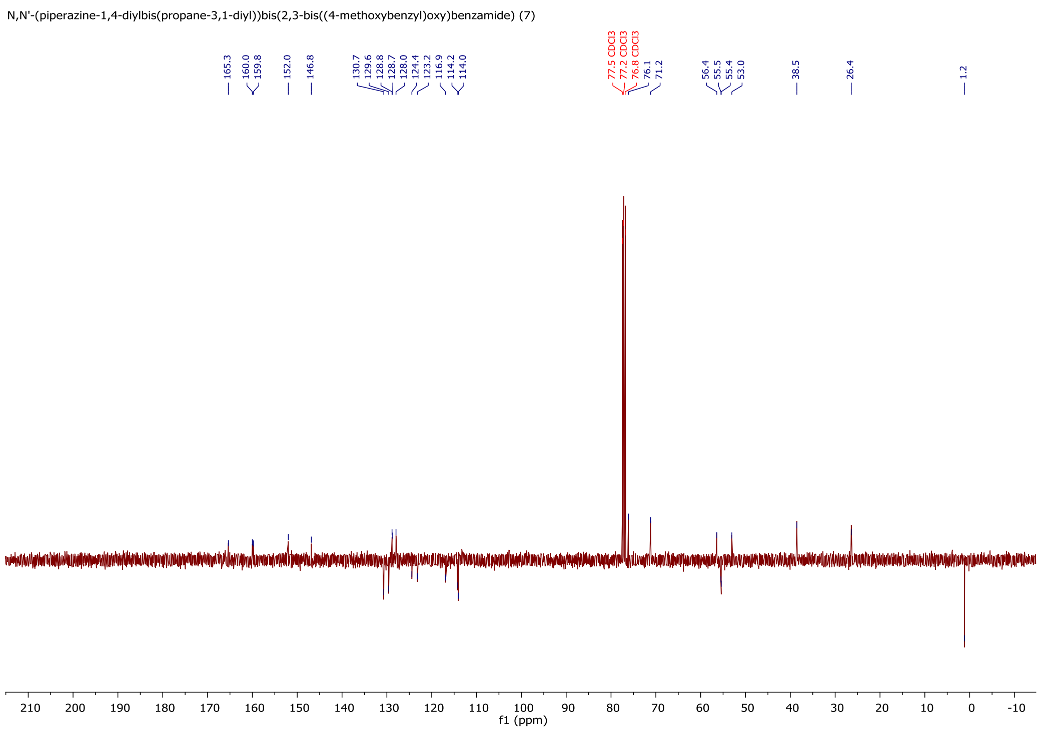

HRMS

Single Mass Analysis
Tolerance = 1.0 PPM / DBE: min = -1.5, max = 150.0
Element prediction: Off
Number of isotope peaks used for i-FIT = 4

Monoisotopic Mass, Even Electron Ions
229 formula(e) evaluated with 1 results within limits (up to 50 closest results for each mass)
Elements Used:
C: 0-60 H: 0-70 N: 0-5 O: 0-15
SD-MS4 48 (0.201) Cm (48:79)

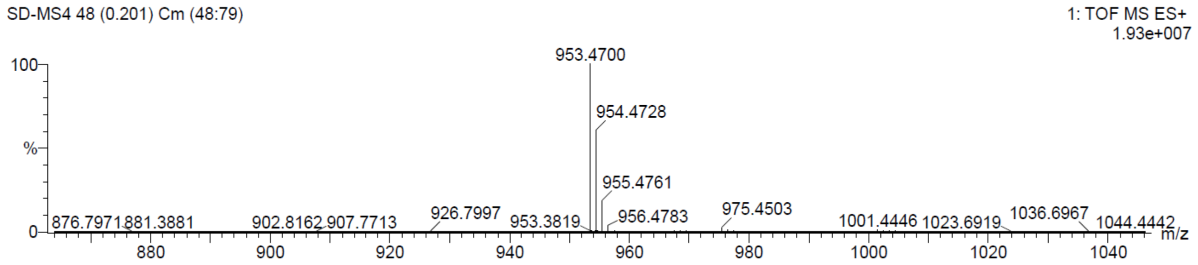

Minimum:				-1.5				
Maximum:		2.0	1.0	150.0				
Mass	Calc. Mass	mDa	PPM	DBE	i-FIT	Norm	Conf(%)	Formula
953.4700	953.4701	-0.1	-0.1	26.5	1324.0	n/a	n/a	C56 H65 N4 O10

1,1′-(piperazine-1,4-diylbis(propane-3,1-diyl))bis(3-((4-methoxybenzyl)oxy)-2-methylpyridin-4(1H)-one) (8)

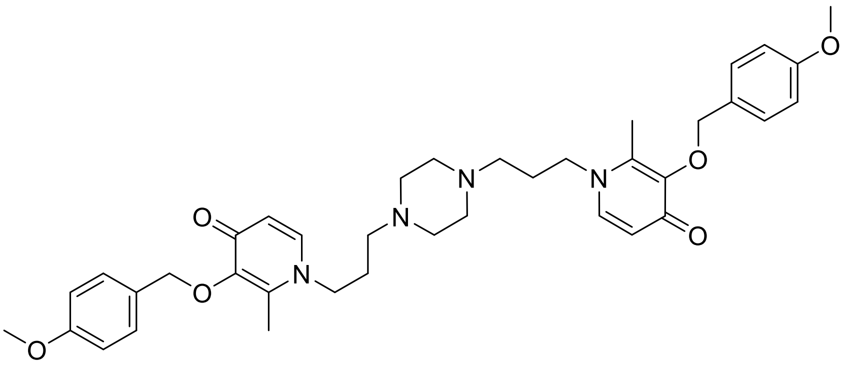

^{1}H NMR

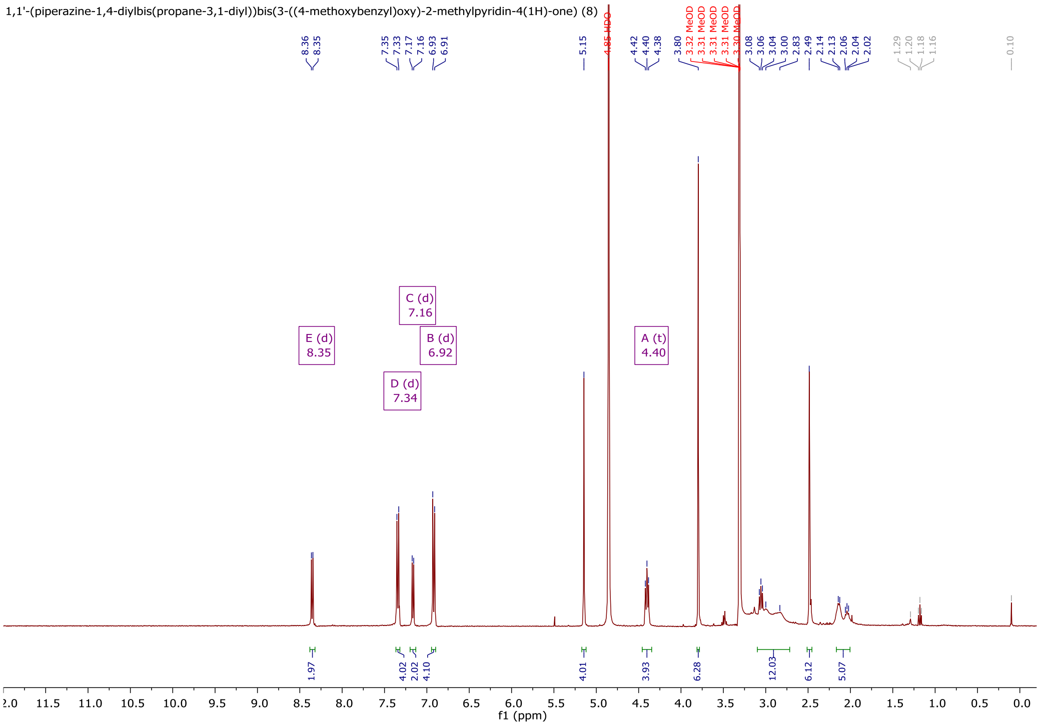

13C NMR

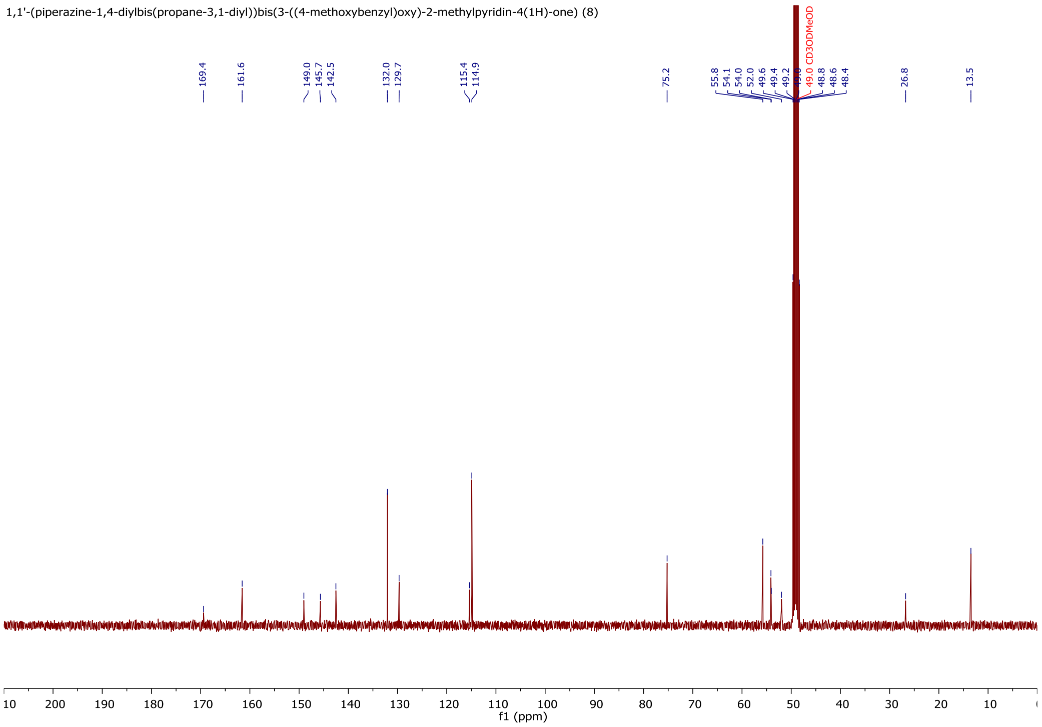

HRMS

Single Mass Analysis
Tolerance = 1.0 PPM / DBE: min = -1.5, max = 150.0
Element prediction: Off
Number of isotope peaks used for i-FIT = 4

Monoisotopic Mass, Even Electron Ions
576 formula(e) evaluated with 1 results within limits (up to 50 closest results for each mass)
Elements Used:
C: 0-60 H: 0-70 N: 0-5 O: 0-15

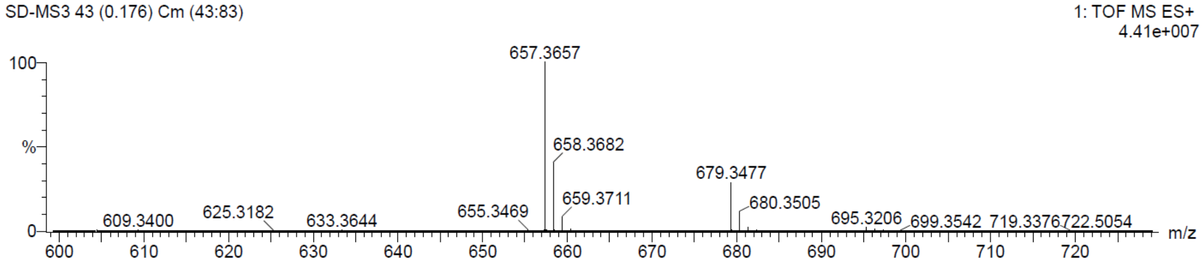

Minimum:				-1.5				
Maximum:		2.0	1.0	150.0				
Mass	Calc. Mass	mDa	PPM	DBE	i-FIT	Norm	Conf(%)	Formula
657.3657	657.3652	0.5	0.8	16.5	1434.7	n/a	n/a	C38 H49 N4 O6

General procedure for the synthesis of gallium complexes Ga(III)-1 and Ga(III)-2. The ligand **1** or **2** (1 eq.) was dissolved in MeOH (10 mL). Tris.HCl was added until pH = 7. The complex Ga(acetylacetonate)$_3$ (26-25 mg, 1.5 eq.) was dissolved in MeOH before addition of Tris.HCl until pH = 7. This mixture was then added in the ligand methanol solution. The corresponding solution was stirred at 40 °C for 3 hours and after at room temperature for 21 hours. Before lyophilization the methanol was evaporated to afford the corresponding gallium complexes-**1** or **2**.

*Ga(III)-**1***. Compound **Ga(III)-1** was obtained from **1** (50mg, 0.105 mmol) according to the general procedure as a pink solid (110 mg).

*Ga(III)-**2***. Compound **Ga(III)-2** was obtained from **2** (50mg, 0.1 mmol) according to the general procedure as a white solid (100 mg).
